# Systematic screening of CTCF binding partners identifies that BHLHE40 regulates CTCF genome-wide distribution and long-range chromatin interactions

**DOI:** 10.1093/nar/gkaa705

**Published:** 2020-09-04

**Authors:** Gongcheng Hu, Xiaotao Dong, Shixin Gong, Yawei Song, Andrew P Hutchins, Hongjie Yao

**Affiliations:** CAS Key Laboratory of Regenerative Biology, Joint School of Life Sciences, State Key Laboratory of Respiratory Disease, Guangzhou Institutes of Biomedicine and Health, Chinese Academy of Sciences, Guangzhou Medical University, Guangzhou 510530, China; Guangdong Provincial Key Laboratory of Stem Cell and Regenerative Medicine, Guangzhou Institutes of Biomedicine and Health, Chinese Academy of Sciences, Guangzhou 510530, China; Bioland Laboratory (Guangzhou Regenerative Medicine and Health GuangDong Laboratory), Guangzhou 510005, China; Institute of Stem Cell and Regeneration, Chinese Academy of Sciences, Beijing 100101, China; University of Chinese Academy of Sciences, Beijing 100049, China; CAS Key Laboratory of Regenerative Biology, Joint School of Life Sciences, State Key Laboratory of Respiratory Disease, Guangzhou Institutes of Biomedicine and Health, Chinese Academy of Sciences, Guangzhou Medical University, Guangzhou 510530, China; Guangdong Provincial Key Laboratory of Stem Cell and Regenerative Medicine, Guangzhou Institutes of Biomedicine and Health, Chinese Academy of Sciences, Guangzhou 510530, China; Bioland Laboratory (Guangzhou Regenerative Medicine and Health GuangDong Laboratory), Guangzhou 510005, China; Institute of Stem Cell and Regeneration, Chinese Academy of Sciences, Beijing 100101, China; University of Chinese Academy of Sciences, Beijing 100049, China; CAS Key Laboratory of Regenerative Biology, Joint School of Life Sciences, State Key Laboratory of Respiratory Disease, Guangzhou Institutes of Biomedicine and Health, Chinese Academy of Sciences, Guangzhou Medical University, Guangzhou 510530, China; Guangdong Provincial Key Laboratory of Stem Cell and Regenerative Medicine, Guangzhou Institutes of Biomedicine and Health, Chinese Academy of Sciences, Guangzhou 510530, China; Bioland Laboratory (Guangzhou Regenerative Medicine and Health GuangDong Laboratory), Guangzhou 510005, China; Institute of Stem Cell and Regeneration, Chinese Academy of Sciences, Beijing 100101, China; University of Chinese Academy of Sciences, Beijing 100049, China; CAS Key Laboratory of Regenerative Biology, Joint School of Life Sciences, State Key Laboratory of Respiratory Disease, Guangzhou Institutes of Biomedicine and Health, Chinese Academy of Sciences, Guangzhou Medical University, Guangzhou 510530, China; Guangdong Provincial Key Laboratory of Stem Cell and Regenerative Medicine, Guangzhou Institutes of Biomedicine and Health, Chinese Academy of Sciences, Guangzhou 510530, China; Bioland Laboratory (Guangzhou Regenerative Medicine and Health GuangDong Laboratory), Guangzhou 510005, China; Institute of Stem Cell and Regeneration, Chinese Academy of Sciences, Beijing 100101, China; Department of Biology, Southern University of Science and Technology, Shenzhen 518055, China; CAS Key Laboratory of Regenerative Biology, Joint School of Life Sciences, State Key Laboratory of Respiratory Disease, Guangzhou Institutes of Biomedicine and Health, Chinese Academy of Sciences, Guangzhou Medical University, Guangzhou 510530, China; Guangdong Provincial Key Laboratory of Stem Cell and Regenerative Medicine, Guangzhou Institutes of Biomedicine and Health, Chinese Academy of Sciences, Guangzhou 510530, China; Bioland Laboratory (Guangzhou Regenerative Medicine and Health GuangDong Laboratory), Guangzhou 510005, China; Institute of Stem Cell and Regeneration, Chinese Academy of Sciences, Beijing 100101, China; University of Chinese Academy of Sciences, Beijing 100049, China

## Abstract

CTCF plays a pivotal role in mediating chromatin interactions, but it does not do so alone. A number of factors have been reported to co-localize with CTCF and regulate CTCF loops, but no comprehensive analysis of binding partners has been performed. This prompted us to identify CTCF loop participants and regulators by co-localization analysis with CTCF. We screened all factors that had ChIP-seq data in humans by co-localization analysis with human super conserved CTCF (hscCTCF) binding sites, and identified many new factors that overlapped with hscCTCF binding sites. Combined with CTCF loop information, we observed that clustered factors could promote CTCF loops. After in-depth mining of each factor, we found that many factors might have the potential to promote CTCF loops. Our data further demonstrated that BHLHE40 affected CTCF loops by regulating CTCF binding. Together, this study revealed that many factors have the potential to participate in or regulate CTCF loops, and discovered a new role for BHLHE40 in modulating CTCF loop formation.

## INTRODUCTION

The eukaryotic genome is organized into three dimensional topologies, which play an important role in gene regulation. It is becoming clear that factors exist to mediate chromosomal contacts, and CTCF has emerged as a leading mediator. CTCF is a ubiquitously expressed, highly conserved vertebrate nuclear protein (1,2), which is crucial for embryonic and adult cell viability (3,4). It has been widely reported to play a critical role in genome organization in bilaterian animals (5–10), and some reports provide direct evidence that targeted disruption of specific CTCF binding motifs could deplete CTCF binding, and result in the disappearance of chromatin interactions (11–15).

While researchers have shown that CTCF can mediate chromatin interactions, how a molecular complex around CTCF is formed remains unclear. CTCF has been postulated to participate in chromatin loops in several ways, including forming sole CTCF–DNA interactions, but the best supported evidence indicates that CTCF forms multimeric complexes by interacting with other proteins (16). CTCF has been reported to bind to SIN3A (17), CHD8 (18), YY1 (19), PARP1 (20), BPTF (21), TAF3 (22), among others, and some of them have been shown to regulate CTCF binding or participate in CTCF loops at specific loci. However, the most widely explored co-factors that co-localize with CTCF are the cohesin complex proteins, consisting of SMC1, SMC3, RAD21 and SA1/2 subunits (23,24). Cohesin is required to stabilize most CTCF-mediated chromatin loops and is critical for CTCF function genome-wide (25–28). To deepen our understanding of how CTCF mediates higher-order chromatin organization, the factors that are involved in CTCF loops need to be explored.

Here, using a computational approach, we identified several well-known factors as well as many new factors that had a high overlap ratio with conserved CTCF binding sites. The more factors that colocalize with CTCF, the stronger loop intensity. These factors might be potential candidates to be involved in CTCF loops, and we go on to experimentally validate one of the novel CTCF binding partners, BHLHE40. Bioinformatics analysis and co-IP experiments indicated that BHLHE40 binding regions overlap with CTCF binding sites, and BHLHE40 forms a protein complex with CTCF. Furthermore, BHLHE40 loss-of-function reduces CTCF binding and disrupts CTCF-mediated long-range chromatin interactions. Taken together, we integrated multiple factor binding and chromatin open states to systemically analyze the features that are related to CTCF looping of DNA, and provide a new description of the organization of CTCF and its binding partners in cells.

## MATERIALS AND METHODS

### Plasmids construction and lentivirus production

Stable knockdown and overexpression cell lines were generated by using the lentiviral system. CTCF CDS (coding sequence) was cloned into a pSin-FLAG vector. shRNAs targeting *BHLHE40* were cloned into a pLKO.1-TRC vector. All the constructs were confirmed by Sanger sequencing, and the shRNA targeting sequences used in this study are described in Supplemental Table S1. For lentivirus production, the lentivirus plasmids were transfected into HEK293T cells and lentivirus supernatant was collected and filtered with 0.45 μM filter at 48 h after transfection.

### Cell culture and lentiviral infection

HeLa-S3 and HEK293T cells were cultured in DMEM (Hyclone) supplemented with 10% fetal bovine serum. HeLa-S3 cells were infected with lentivirus. After 48 h infection, puromycin (2 μg/mL) was added to the medium to select positively infected cells.

### Antibodies

The following antibodies were used in this study: mouse anti-ACTIN antibody (Abcam, ab3280), rabbit anti-CTCF antibody (Millipore, 07-729), anti-BIOTIN HRP-linked antibody (Cell Signaling Technology, #7075) for western blot, rabbit anti-BHLHE40 antibody (Novus, NB100-1800) for western blot and ChIP experiments, Flag M2 beads (Sigma, M8823) for Flag co-IP, Dynabeads M-280 Streptavidin (Thermo Fisher Scientific, 11205D) for BIOTIN ChIP-seq, rabbit anti-CTCF antibody (Active Motif, 61311) for CTCF HiChIP.

### Western blot

The cells were resuspended and sonicated in RIPA buffer (0.1% SDS, 1% Triton X-100, 150 mM KCl, 50 mM Tris–HCl [pH 7.4], 1 mM EDTA, 1 mM PMSF and 1× protease inhibitor cocktails). Total soluble proteins were obtained by centrifugation at 12 000 rpm for 10 min. Samples were separated on SDS-PAGE gel and transferred onto a PVDF membrane (Millipore). The PVDF membrane was blocked with 5% milk in TBS-T (TBS with 0.05% Tween-20). Immunoblot analysis was performed with the indicated antibodies.

### Co-IP experiments

To verify the interaction between CTCF and BHLHE40, nuclear extract (NE) of HeLa-S3 cells overexpressing FLAG tagged CTCF or BIOTIN tagged BHLHE40 was used. To make soluble nuclear extract, cells were washed once and swollen in hypotonic buffer (10 mM Tris [pH 7.4], 10 mM KCl, 1.5 mM MgCl_2_, 1 mM PMSF and 1 × protease inhibitor cocktails) for 10 min on ice followed by homogenization eight times with a loose pestle. Nuclei were centrifuged at 2000 × g for 10 min at 4°C, then the supernatant was discarded. Nuclear pellets were resuspended in 0.5 volume low salt buffer (20 mM Tris [pH 7.4], 20 mM KCl, 25% glycerol, 1.5 mM MgCl_2_, 0.2 mM EDTA, 1 mM PMSF and 1× protease inhibitor cocktails) and homogenized six times with a loose pestle. High salt buffer (20 mM Tris [pH 7.4], 1.2 M KCl, 25% glycerol, 1.5 mM MgCl_2_, 0.2 mM EDTA, 1 mM PMSF and 1× protease inhibitor cocktails) was slowly added. Nuclei were rotated for 30 min and centrifuged at 14 000 × g for 15 min at 4°C. After that, the supernatant was collected. The insoluble material was re-suspended in 0.5 volume TGME buffer (50 mM Tris [pH 7.9], 25% glycerol, 5 mM MgCl_2_, 0.1 mM EDTA, 1 mM PMSF and 1× protease inhibitor cocktails), homogenized with a loose pestle at least 20 times. Supernatant was collected after centrifugation at 14 000 × g for 15 min at 4°C and combined with previous supernatant. Then nuclear extract was incubated with Flag M2 or Dynabeads M-280 Streptavidin beads with rotation at 4°C overnight. After three washes with IP wash buffer (20 mM Tris [pH 7.9], 0.1 M KCl, 5 mM MgCl_2_, 10% glycerol, 0.1% Tween-20, 1 mM PMSF and 1× protease inhibitor cocktails), proteins bound on the M2 or Dynabeads M-280 Streptavidin beads were boiled with 1× SDS loading buffer for 10 min. And the eluted bound proteins were analyzed by western blot.

### RT-qPCR

Total RNA was isolated with TRIzol and cDNAs were synthesized by using Reverse Transcriptase (TOYOBO). Quantitative real-time PCR was performed with SYBR green mixture (Genstar) on a CFX Real-Time System (Bio-Rad). The primers used in the RT-qPCR assays are listed in Supplemental Table S1.

### ChIA-PET analysis

CTCF ChIA-PET data was downloaded from GEO database and analyzed with ChIA-PET2 software (29) using the hg19 genome. For A/B linker CTCF ChIA-PET data in K562 and MCF7 cells, the parameters “-A GTTGGATAAG -B GTTGGAATGT -m 0 -e 1 -k 0 -t 10 -d 1 -M ‘-q 0.05' -Q 30 -C 1 -S 100 -E 500 -l 15" were used. For bridge linker ChIA-PET data in GM12878 and HeLa-S3 cells, the parameters “-A ACGCGATATCTTATC -B AGTCAGATAAGATAT -m 1 -e 1 -k 0 -t 10 -d 0 -M ‘-q 0.05' -Q 30 -C 1 -S 100 -E 500 -l 15" were used. ChIA-PET peaks were adjusted to 500 bp around the peak summit.

### Identification of human super conserved CTCF binding sites

Conserved peaks from four ChIA-PET data were overlapped with ENCODE CTCF peaks, which were extracted from wgEncodeRegTfbsCellsV3.tab, peak overlapping was performed by bedtools intersect (v2.25) (30) to identify human super conserved CTCF binding sites (hscCTCF binding sites) (Supplemental Table S2). These hscCTCF binding sites and genome-wide CTCF binding sites identified in the above four cell types were annotated with homer (31) annotatePeaks.pl tool against the hg19 genome.

### Uniform analysis of co-factor ChIP-seq data

All ChIP-seq data collected from GEO database were first transformed to fastq files using fastq-dump (v2.8.2). Raw reads were subjected to Trim Galore (v0.4.4) to trim adaptors and low-quality reads. Trimmed reads were aligned to the hg19 human genome assembly using bowtie2 (v2.2.5) (32) with the parameters “–very-sensitive –end-to-end –no-unal". Then the aligned reads with a MAPQ >30 were selected by samtools (v1.2) (33), and duplicate reads were removed by picard tools (v1.90). For single-end data, phantom tool was used to calculate fragment length, which was used in the following peak discovering process. Peak calling was performed with MACS2 (v2.1.0) (34) using the parameter “-q 0.01", any peak overlapped with blacklist regions (ENCODE DAC), or in chrM, chrY was removed. The datasets with deduplicated reads less than 5 million, or with no peaks using our criteria were filtered out. For each factor with replicate experiments, we chose the data with maximum number of peaks.

### Stringent overlap analysis between protein factor and hscCTCF or CTCF binding sites in the genome

Peaks from human protein factor ChIP-seq data, hscCTCF binding sites and genome-wide CTCF binding sites were all adjusted to 200 bp by extending 100 bp for each direction from the peak summit. Peak cobinding analysis has been performed by using bedtools intersect. By overlapping with hscCTCF binding sites, at most top 100,000 peaks were extracted to avoid false positive results.

### Determining CTCF candidate regulatory modules

Candidate regulatory modules (CRMs) were generated by merging ChIP-seq peak data, similar to the procedure in a previous study (35). Generally, the binding sites from all transcription factors were merged by using bedtools merge function and the finally merged regions were called CRMs. To minimize excessive peak overlap, which may cause nearby CRMs to merge, we adjusted all peak widths to 100, 150 and 200 bp based on their summits, and then the adjusted peak regions were merged to generate CRMs. CRMs containing CTCF peaks were selected as CTCF CRMs, and the width distribution of CTCF CRMs was evaluated. When merging 100 bp peak regions together, the width of 90% CTCF CRMs was in the interval of 0.3-2.1 kb. When 150 bp, the range was 0.4-2.6 kb. And when 200 bp, the range was 0.5-3.2 kb. Since the regions produced by merging 100 bp peak regions were already long enough, these CTCF CRMs were chosen. For CTCF CRMs in each cell type, one CRM only containing one CTCF binding site in this cell type was chosen for further analysis.

### Correlation analysis between DNase signal, ChIP enrichment and the strength of CTCF-mediated loops

To evaluate the correlation between ChIP enrichment and CTCF looping, CTCF loop anchor regions identified in CTCF ChIA-PET data were used, if two or more CTCF binding sites were contained in the same loop anchor region, these binding sites were filtered out, which ensured that each loop anchor corresponded to a unique CTCF binding site. CTCF loop strength mediated by each anchor was calculated by collecting all loop PET tags mediated by this anchor.

The DNase signal, factor binding strength or ChIP enrichment of histone markers were calculated as below. The coverage of their peak regions was first extracted using bedtools coverage, then normalized as log_2_(coverage × 10^9^/peak length × 10^6^). CTCF loop strength was also transformed and expressed as log_2_. The Pearson correlation between them was calculated in R using the cor() function. When the peak numbers were <1000 or the *P*-value from Pearson correlation was larger than 0.01, the correlation between them was considered as unreliable, and −log_10_(*P*-value) was set to zero. *P*-value is further adjusted for multiple comparisons with Bonferroni correction.

### Paired factor analysis at paired loop anchors

Since each chromatin loop is connected with two chromatin anchor regions, we hypothesized that the factors binding at the two anchors of each loop might form a complex to promote loop formation. We combined any two factors from each paired loop anchor as a factor pair, representing all possible direct or indirect protein interactions, and then gave each pair a score, which was the original loop strength in the cells, and finally pooled all factor pairs together with the scores summed up for the same factor pair. Factor pairs with their scores were further integrated into a network in cytoscape software (36). The thicker edge between two factors represents a higher factor pairing frequency.

### ChIP-seq

ChIP experiments were performed as previously described (37). Briefly, 1 × 10^7^ cells were crosslinked with 1% formaldehyde for 10 min at room temperature, then the reaction was stopped by adding glycine (final concentration, 0.125 M). Crosslinked cells were lysed in ChIP SDS lysis buffer (1% SDS, 10 mM EDTA, 50 mM Tris-HCl [pH 8.0]) containing 1× protease inhibitor cocktail and PMSF, then sonicated to achieve a chromatin size of 200-400 bp. After sonication, the supernatant was diluted with IP buffer and then co-incubated with protein A and protein G dynabeads (1:1 mix) and the indicated antibodies at 4°C overnight with rotation. Antibody bound DNA was subsequently washed with low salt wash buffer (0.1% SDS, 1% Triton X-100, 2 mM EDTA, 20 mM Tris-HCl [pH 8.0], 150 mM NaCl), high salt wash buffer (0.1% SDS, 1% Triton X-100, 2 mM EDTA, 20 mM Tris-HCl [pH 8.0], 500 mM NaCl), LiCl wash buffer (0.25 M LiCl, 1% IGEPAL-CA630, 1% deoxycholic acid, 1 mM EDTA, 10 mM Tris-HCl [pH 8.0]) once, respectively, and then TE wash buffer (10 mM Tris-HCl [pH 8.0], 1 mM EDTA) twice. ChIPed DNA was reverse-crosslinked and purified for DNA library construction followed by sequencing or ChIP-qPCR analysis. Primers used for ChIP-qPCR were listed in Supplemental Table S1. Both BHLHE40 and CTCF ChIP-seq experiments have two biological replicates. We further performed BIOTIN ChIP-seq for BIOTIN-BHLHE40 to validate BHLHE40 ChIP-seq. Adaptor oligonucleotides and primer sequences from Illumina were used for library construction and amplification. ChIP-seq libraries were constructed using the VAHTS™ Universal DNA Library Prep Kit for Illumina^®^ V2. After PCR library amplification, size selection of adaptor-ligated DNA was performed using Agecourt AMPure XP Beads (Beckman Coulter). The libraries were diluted at a proper concentration for sequencing and finally sequenced on HiSeq X-Ten (Annoroad Gene Technology Co., Ltd.).

### ChIP-seq data analysis

Raw reads are subjected to Trim Galore to remove adaptors and low-quality reads, then trimmed reads were mapped to female hg19 genome using bowtie2 with parameters “--very-sensitive --end-to-end --no-unal", proper aligned and high quality mapped (MAPQ >30) reads were selected and reads in blacklist region or chrM were further removed, duplicates were removed using Picard. For CTCF ChIP-seq in HeLa-S3 cells with control shRNA, BHLHE40-depleted cells, unique high-quality reads were uniformly subsampled to 20 million reads using set.seed (9999) and sample function in R. Peaks were called by MACS2 with the parameter “-q 0.01". For BHLHE40 and BIOTIN-BHLHE40 ChIP-seq data, peaks were called by MACS2 with default parameters “-q 0.05". For all data, normalized signal tracks were generated by using bamCoverage from deeptools with parameter “--normalizeUsing RPGC". Differential CTCF binding sites were identified using Diffbind package (38), binding sites with log_2_(fold_change) >1 and *P*-value <0.01 were considered as significantly differential binding sites, which were used for further analysis. Results from DiffBind were listed in Supplemental Table S7.

### RNA-seq data analysis

All RNA-seq experiments have two biological replicates. Raw reads were firstly trimmed to remove the adaptors and low-quality reads by using Trim Galore, then mapped to human genome (hg19 sourced from UCSC genome browser) using STAR (v2.5.2a) (39), gene expression levels were quantified as read counts generated by RSEM (v1.2.22) (40), with default settings. Raw tag counts were normalized for GC content using EDASeq (v2.8.0) (41). For a gene to be regarded as expressed, the gene must have at least 10 normalized tags in any two samples. Differential gene expression was analyzed with DESeq2 (v1.10.1) (42). Gene expression was considered as changing if it was significantly different (*q*-value <0.05) and with fold change >2. These differentially expressed genes were listed in Supplemental Table S8.

### HiChIP experiments

The HiChIP protocol was performed as previously described (43,44) with some modifications. In brief, up to 15 million crosslinked cells were washed in 500 μL of ice-cold Hi-C lysis buffer (10 mM Tris–HCl [pH 7.5], 10 mM NaCl, 0.2% NP-40, 1× protease inhibitor cocktails) twice. The nuclei pellet was resuspended in 100 μL of 0.5% SDS and incubated at 62°C for 10 min with no shaking or rotation and then the reaction was quenched with Triton X-100 at 37°C for 15 min. *Mbo*I restriction enzyme (NEB, R0147) was added at 37°C for 2 h to digest the nuclei, and heat inactivated at 62°C for 20 min. After filling in the restriction fragment overhangs and marking the DNA ends with biotin, *in situ* contact was generated by proximity ligation. The nuclei with *in situ* generated contacts were pelleted at 2500 × g for 5 min at room temperature and the nuclear pellet was resuspended in nuclear lysis buffer (50 mM Tris [pH 7.5], 10 mM EDTA, 1% SDS, 1× protease inhibitor cocktails) for sonication, and clarified by centrifugation at 16 100 × g at 4°C for 15 min. The clarified samples were transferred to a new tube and diluted with ChIP dilution buffer (0.01% SDS, 1.1% Triton X-100, 1.2 mM EDTA, 16.7 mM Tris [pH 7.5], 167 mM NaCl) for ChIP procedures. ChIPed DNA was quantified by Qubit (Thermo Fisher) to estimate the amount of Tn5 (Illumina) needed to generate libraries at the correct size distribution. 150 ng of ChIPed DNA was taken into the biotin capture step and tagmented with Tn5. Finally, the tagmented DNA containing beads was PCR amplified and size selected with AMPure XP beads (Beckman). After size selection, libraries were quantified with qPCR against Illumina primers and/or bioanalyzer. Libraries were paired-end sequenced with read lengths of 150 bp.

### HiChIP data analysis

Paired-end HiChIP raw data were subjected to Trim Galore to remove adaptors. Trimmed reads were aligned to the hg19 genome using the HiC-Pro software (45), with default settings except that reads were assigned to *Mbo*I restriction fragments. Valid reads from HiC-Pro results were further processed to call loops with hichipper (46). Normalized genome-wide signal coverage files were generated and transformed into bigwig files by MACS2 and bedGraphToBigWig, then HiChIP correlation analysis was performed by deeptools (47). And ICE normalized matrix in 5 or 10 kb resolution from HiC-Pro results was used to draw heatmaps. CTCF HiChIP replicate data was combined to identify loops. Loops were then corrected with mango (48), and loops with FDR <0.05 were selected. We further extracted loops with either anchor region containing CTCF peaks, which were CTCF peaks merged from CTCF ChIP-seq data. These selected loops were listed in Supplemental Table S9 and used for further analysis. For differential loop identification, we filtered out the loop which the maximum loop number in control and BHLHE40-depleted cells was <2, then calculated the fold change after adding 1 to each loop count. The loops with change >2-fold were considered as differential loops.

### Identification of enhancer-promoter loops

#### Loop identification from Hi-C data

Hi-C data from HeLa-S3 cells with two replicates was downloaded from GSE133462 (GSM3909686 and GSM3909709) (49). Sra data format was transformed into fastq by fastq-dump tool. The raw reads were trimmed with *Dpn*II restriction sequence “GATC" with homerTools, and then mapped to human hg19 assembly using bowtie2 with parameters “--very-sensitive --end-to-end --no-unal". After mapping, two replicates were combined. The HOMER program makeTagDirectory was first used to create tag directories with “tbp 1" parameter. Data was further processed by HOMER in order to remove small fragments and self-ligations using makeTagDirectory with the following options: -removePEbg -restrictionSite GATC -both -removeSelfLigation -removeSpikes 10000 5. HOMER program analyzeHiC was used to obtain significant loops at a 10 kb resolution with default parameters. Loops with FDR <0.01 were used for further analysis.

#### Identification of active promoter and enhancer regions

TxDb.Hsapiens.UCSC.hg19.knownGene package has been used to extract ± 500 bp regions of the TSS of all transcripts as candidate promoter regions. Previous defined regions for EnhG1, EnhG2, EnhA1, EnhA2, EnhWk in HeLa-S3 cells (50) were combined as candidate enhancer regions. To identify active promoters and enhancers, we analyzed GRO-seq data from HeLa-S3 cells (GSM3100195) (51). Raw data were transformed into fastq files, and adaptor and polyA were removed. Then the data was mapped to hg19 genome using bowtie2. Mapped reads were processed with groHMM package (52) with parameters “LtProbB -100,UTS 15". Candidate promoters and enhancers, which could intersect with transcript regions generated from GRO-seq data, were selected as active promoters and enhancers.

#### Enhancer–promoter loop extraction

Loops were annotated to the promoter and enhancer regions with GenomicInteractions package (53), then promoter-enhancer loops were selected. After that, 11 083 EP loops have been obtained. As the resolution for loop identification is 10 kb, the anchor region might contain multiple enhancers or gene promoters. From the identified EP loops, all EP loop combinations were generated and totally 30 685 putative EP loops were chosen for the subsequent analysis.

## RESULTS

### Identification of putative CTCF co-factors

**Figure 1.**
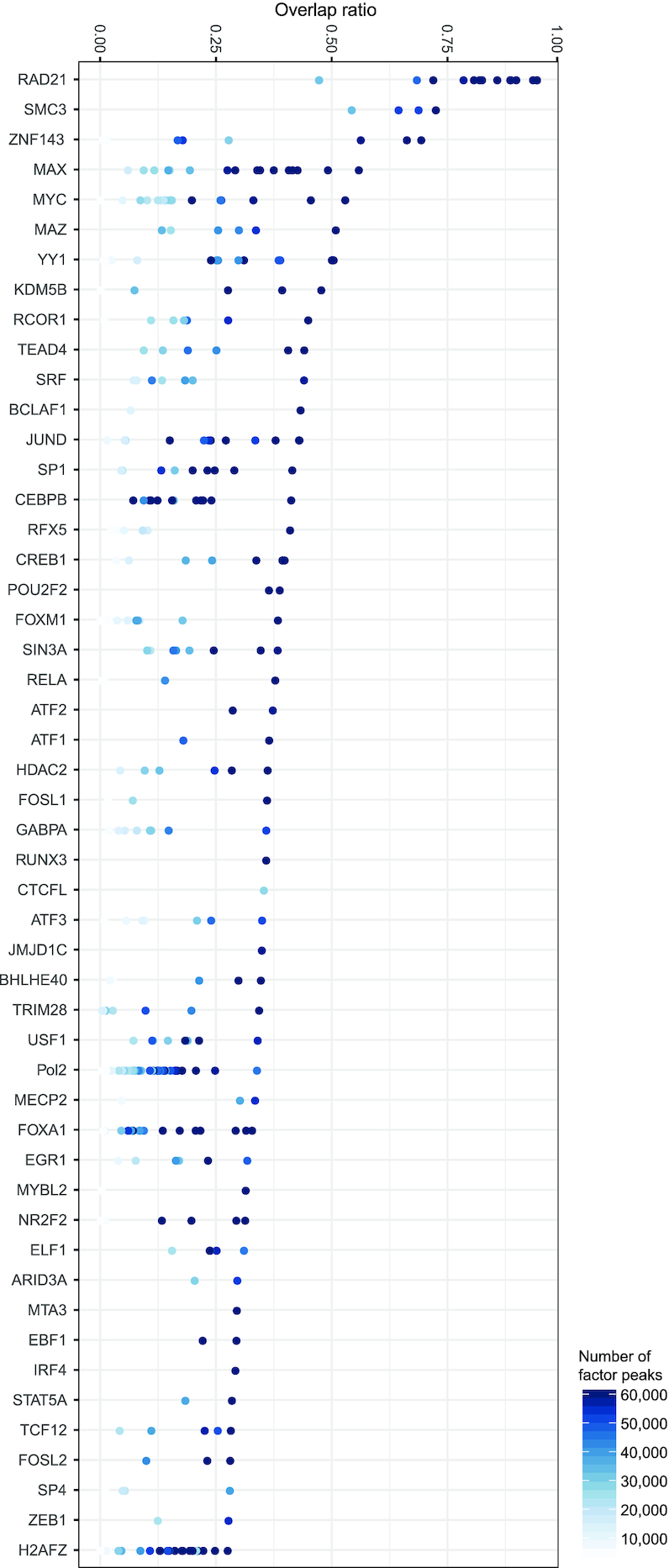
The top 50 factors ranked by maximum overlap ratio between these factors and CTCF. The overlap ratio of each factor with CTCF was calculated by using the overlapped CTCF binding sites divided by the total number of CTCF binding sites. Each dot represents a ChIP-seq result for each factor in one of the ENCODE cell lines.

To identify factors participating in CTCF-mediated chromatin interactions, we collected CTCF co-localized factors using ChIP-seq data for chromatin-associated proteins. CTCF ChIP-seq data from the ENCODE project, which contains 99 CTCF ChIP-seq datasets from 70 cell types or treatments, were utilized (54). We then collected 1306 ChIP-seq data for 431 protein factors (Supplemental Table S3), including transcription factors, histone variants, and histone-modifying enzymes, in 23 cell lines that also have CTCF ChIP-seq data in the same cell type. These ChIP-seq data were uniformly reanalyzed (see Materials and Methods) and the resulting peaks were overlapped with CTCF binding sites in the same cell line to measure the fraction of CTCF sites that are co-bound by the indicated factors. Sorting the data according to the maximum overlap ratio for each factor (Figure 1) revealed many known CTCF co-factors, including cohesin subunits (RAD21, SMC3), histone demethylase KDM5B, and transcription factors YY1 and ZNF143 (19,23,25,55,56), indicating our bioinformatic analysis is reliable. We additionally identified many other factors with a high overlap ratio that have not previously been implicated in CTCF function. The list of factors that are often co-bound with CTCF is thus a rich resource to explore CTCF looping participants or regulators.

Except the ChIP-seq data for the above explored factors, there is still a large amount of untapped ChIP-seq data (57), which might contain new co-localized CTCF co-factors. However, a desirable prerequisite for the analysis of co-factor co-localization is that the ChIP-seq experiments should be conducted in the same cell type under the same conditions. However, most data do not match such conditions. To overcome this problem, we took advantage of a unique property of CTCF, which is its surprisingly cell type-independent pattern of binding, leading to a large number of highly conserved binding sites in widely divergent cells and tissues (58,59). These conserved CTCF binding sites could then be compared to other co-factor data, even data coming from different cell types, and so maximize the identification of potential binding partners.

**Figure 2.**
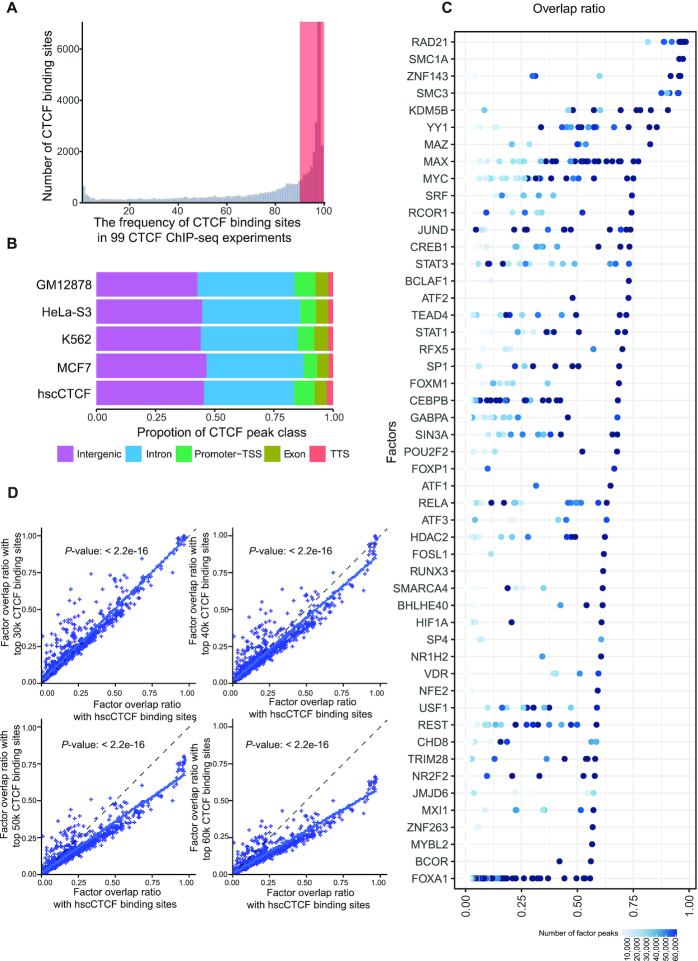
Identification of human super conserved CTCF (hscCTCF) binding sites. (**A**) Bar plot showing the distribution of conserved CTCF binding sites derived from four CTCF ChIA-PET datasets in 99 CTCF ChIP-seq datasets. (**B**) The genomic distribution of hscCTCF binding sites and genome-wide CTCF binding sites for the indicated cell lines. Genomic features are color-coded in the legend bar. The x-axis shows the cumulative percentage of genomic occupancy of each feature. (**C**) The top 50 protein factors ranked by each factor's maximum overlap ratio with hscCTCF sites. The overlap ratio for each factor with hscCTCF was calculated by using the overlapped hscCTCF binding sites divided by total hscCTCF binding sites. Each dot represents a ChIP dataset. (**D**) Scatter plots showing the relationship between the factor overlap ratio generated using hscCTCF binding sites and the ratio generated using the top 30 000, 40 000, 50 000 and 60 000 CTCF binding sites.

We prepared conserved CTCF binding sites by integrating all CTCF ChIP-seq data from the ENCODE project with conserved CTCF ChIA-PET peaks from different cell lines (GM12878, HeLa-S3, K562 and MCF7) to ensure that the binding sites could mediate chromatin loops (Supplemental Figure S1A and B). In total, we identified 20 875 CTCF binding sites (Figure 2A), which we termed “human super conserved CTCF” (hscCTCF) binding sites. The binding strength of hscCTCF is stronger than that of all CTCF binding sites in the selected cell lines (Supplemental Figure S2), which is consistent with previous reports (60,61). The genomic distribution of these peaks indicated no substantial bias compared to the total set of peaks in the selected cell lines (Figure 2B). ChIP-seq datasets for human transcription factors in the GEO database (62) were collected, analyzed and filtered with our pipeline and criteria. 3438 ChIP-seq datasets for 1057 factors were used (Supplemental Table S4) and overlapping analysis was performed. Though all the overlap ratio was relatively higher than previous results, topmost CTCF co-occupied factors identified using total CTCF binding sites were still highly ranked (Figure 2C and Supplemental Table S5). Further, we compared the overlap ratio generated with total CTCF sites with that generated with hscCTCF sites. In order to avoid overlap ratio differences caused by the different total CTCF peak number in any one cell type (Supplemental Figure S1C), we trimmed the top 30 000, 40 000, 50 000, 60 000, or all CTCF binding sites by their binding strength to represent genome-wide CTCF binding sites before performing overlapping analysis. We observed that the overlap ratio generated using hscCTCF binding sites had a good linear relationship with the overlap ratio generated by using whole genome CTCF binding sites (Figure 2D), which suggested that overlap ratio using hscCTCF binding sites can, to a large extent, represent the overlap ratio from the full list of CTCF binding sites. And we further found that the overlap ratio of some of the transcription factors with hscCTCF binding sites was higher than that with all CTCF sites, for example SMARCA4, which has been reported to be a CTCF interacting protein (63). Therefore, our data suggested that using hscCTCF binding sites provides a reliable way to evaluate the co-localization between specific protein factors and CTCF.

### Comprehensive analyses of features related to CTCF loops

**Figure 3.**
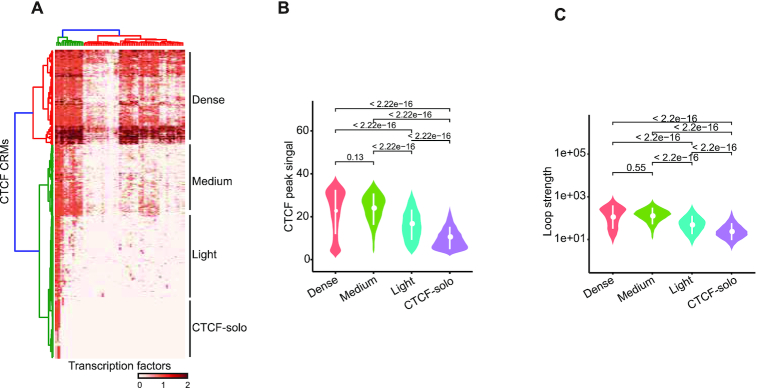
Genomic features of CTCF loop anchors. (**A**) Heatmap displaying the density of CTCF co-localized factors grouped by hierarchical clustering. Dense, medium, light and CTCF-solo binding sites represent different frequency of factor co-binding, respectively. CTCF co-localized factors used in the heatmap were listed in Supplemental Table S6. (**B**) Violin plot showing the distribution of CTCF peak signal which is the value of seventh column in the narrowPeak file generated by MACS2. (**C**) The distribution of CTCF loop strength among four CTCF binding groups with log_10_ scale on y axis. Loop strength is represented as the sum of the anchor mediated all loop PET tag counts. *P* value in (**B**) and (**C**) was calculated using Wilcoxon rank sum test.

To further explore the relationship between CTCF co-binding factors and CTCF loops, we defined CTCF candidate regulatory modules (CRMs) (see Materials and Methods), similar to a previous study (34), which could capture all possible CTCF co-localized factors in four CTCF ChIA-PET cell lines relative to peak overlap analysis. Briefly, we used all co-factor peaks and uniformly resized the peak width to 100 bp, and merged them into a superset of regulatory regions. For each cell type, the regions that contained CTCF and cohesin and overlapped with CTCF ChIA-PET peaks were extracted as CTCF CRMs. Many identified factors were confirmed to co-localize with CTCF (Supplemental Figure S3). Based on hierarchical clustering, we categorized CTCF binding sites into four groups and defined them as “dense", “medium", “light" and “CTCF-solo" binding patterns based on the frequency of factor co-binding (Figure 3A and Supplemental Figure S4A). We evaluated CTCF binding strength and loop strength among different groups and found that the dense and medium groups had stronger CTCF binding, whilst the light and CTCF-solo groups showed significantly reduced CTCF signal (Figure 3B and Supplemental Figure S4B). CTCF loop strength among different groups showed similar results (Figure 3C and Supplemental Figure S4C). These results indicated that the regions that had more co-localized co-factors possessed stronger CTCF binding and formed stronger chromatin loops, suggesting the presence of CTCF-loop promoting factors.

### Individual factor analysis reveals potential loop-promoting factors

**Figure 4.**
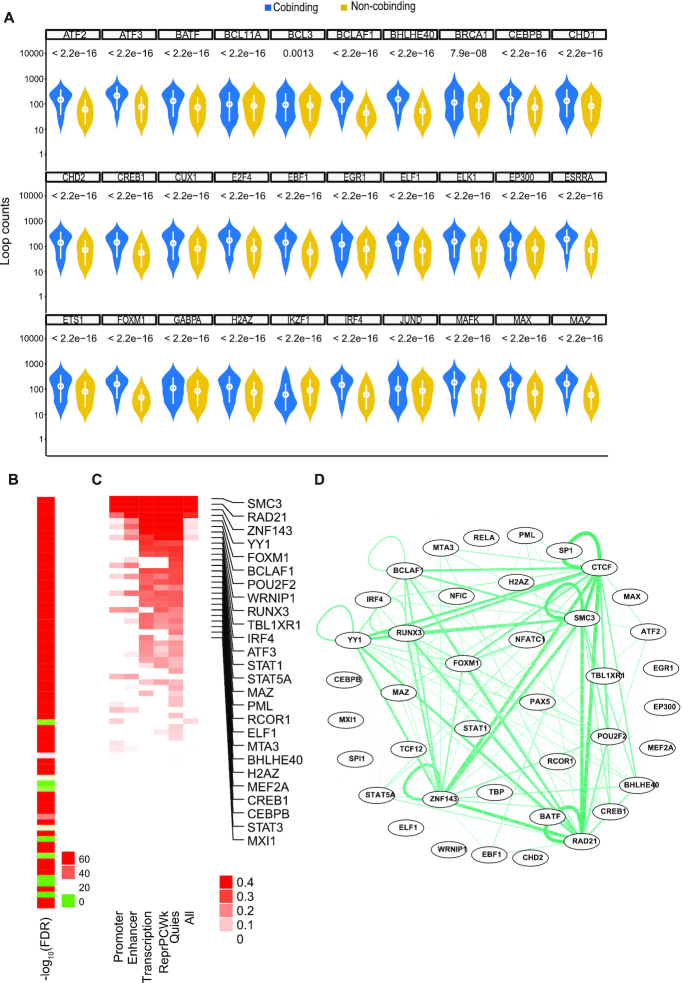
Individual factor analysis reveals potential loop-correlated factors. (**A**) Examples of CTCF loop strength distribution between factor cobinding and non-cobinding regions for each protein factor in GM12878 cells. Factors were shown alphabetically. Y axis is log_10_ scaled. (**B**) Heatmap showing –log_10_(FDR) of CTCF loop strength between factor cobinding sites and factor non-cobinding sites for each protein factor. (**C**) Heatmap showing the correlation between normalized factor binding strength and log_2_ transformed CTCF loop strength. Factors were listed by maximum correlation, and the factor with maximum correlation >0.2 was selected. (**D**) Network visualization displaying the frequency of factor pair appearance in paired loop anchors. FDR value in (A) and (B) was calculated using Wilcoxon rank sum test and adjusted for multiple comparisons using Bonferroni correction.

Given that the CTCF co-localized factors might promote CTCF loops, we explored the potential roles of these factors in the four cell types in which CTCF-mediated chromatin loops have been described (64). Furthermore, two strategies were utilized to evaluate factor loop-promoting capacity. First, for each factor, we divided CTCF binding sites into factor cobinding and non-cobinding groups. We hypothesized if a co-factor of CTCF could prompt looping, then cobinding regions of CTCF with this co-factor might mediate stronger loop formation than non-cobinding regions. Comparing loop strength between co-binding and non-cobinding regions revealed that loop strength distribution for the majority of factors in the cobinding regions was significantly stronger than that in non-co-binding regions (Figure 4A). After removing the factors that did not fulfill the above criterion, a false discovery-rate (FDR) was calculated to aid comparison (Figure 4B, Supplemental Figure S5A, 6A and 7A). We further filtered out factors with –log_10_(FDR) <20 and analyzed the relationship between DNA binding and loop strength for the remaining co-factors. As expected, the cohesin subunit RAD21 emerged as the co-factor with the highest score, as it is critical for CTCF loops (25,26) (Supplemental Figure S8A and B). Considering that these protein factors might exert specific functions when bound to different genomic regions, we annotated CTCF binding sites with 18 chromatin states data from the Roadmap Epigenome project (50). CTCF binding sites could be divided into five categories: promoters, enhancers, transcription, reprPCWk (weak repressed Polycomb regions) and mainly quies (quiescent) regions (Supplemental Figure S9). Thereby, we divided the factor-binding regions into these five groups and calculated the correlation for each group. The binding strength of previously reported CTCF co-factors, such as the cohesin subunits, ZNF143 and YY1, have a good correlation with CTCF-mediated loop strength (Figure 4C, Supplemental Figures S5B, S6B and S7B). Importantly, we also noticed that many other co-factors have a significant correlation with CTCF loop counts. Therefore, we concluded that those cofactors which had both a significant FDR and good correlation with CTCF are potential CTCF loop-promoting factors.

Potentially, CTCF co-factors located at both anchors of a chromatin loop are more likely to result in the promotion of chromatin loops. We next sought out potential factor pairs or complexes (See Methods). This strategy does not depend on correlation analysis, which might be biased by the value of looping strength or CTCF binding strength. Our data showed that many factors such as CTCF, cohesin subunits, MAX, MAZ and BHLHE40 frequently existed at both of the two loop anchors (Figure 4D, Supplemental Figures S5C, S6C and S7C). These data suggest that they might form a complex together with CTCF and participate in CTCF loops.

### BHLHE40 regulates CTCF mediated chromatin interactions

**Figure 5.**
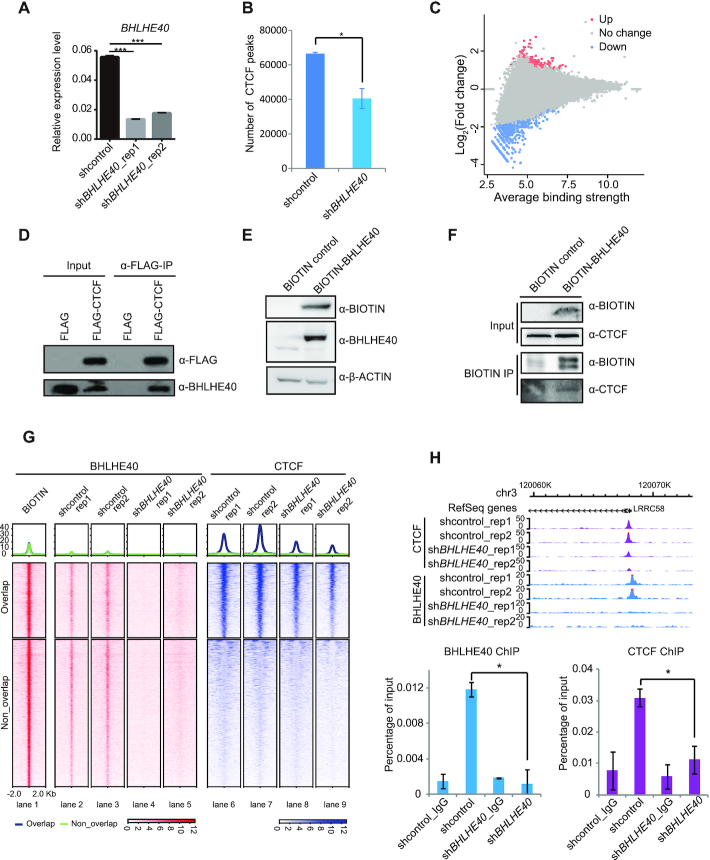
BHLHE40 influences the genomic binding of CTCF. (**A**) Bar plot showing shRNA knockdown efficiency assessed by RT-qPCR. Results are from three biological replicates. Data are represented as mean ± SEM. *** *P* <0.001. *P*-value is calculated using two-tailed Student's *t* test. (**B**) The number of CTCF peaks in control shRNA and BHLHE40-depleted HeLa-S3 cells. (**C**) Scatter plot showing the CTCF binding difference in control shRNA and BHLHE40-depleted HeLa-S3 cells. (**D**) Immunoprecipitation from HeLa-S3 nuclear extracts with FLAG antibody for FLAG-tagged CTCF. Bound proteins were resolved on SDS-PAGE and detected by western blotting for the indicated antigens. (**E**) Western blot results verifying the overexpression of BIOTIN-tagged BHLHE40 in HeLa-S3 cells. (**F**) Detection of the interaction between BHLHE40 and CTCF by BIOTIN IP experiments using soluble nuclear extracts of BIOTIN-tagged BHLHE40 HeLa-S3 cells. (**G**) Normalized tag density heatmap for BHLHE40 binding sites with corresponded CTCF binding sites. 2 kb regions are shown centered on the midpoints of the BHLHE40 peaks. (**H**) Screenshot from the WashU epigenome browser showing a BHLHE40/CTCF overlap binding sites at the promoter of *LRRC58*. ChIP-qPCR results (lower bar charts) show the decrease of BHLHE40 and CTCF enrichment following sh*BHLHE40* treatment. Data are from three biological replicates and represented as mean ± SEM. *P <0.05. *P*-value is calculated by using two-tailed Student's *t* test.

To validate our computational analysis for CTCF co-binding factors, we selected BHLHE40 for further study. We first knocked down BHLHE40 using specific shRNAs (Figure 5A), and then performed CTCF ChIP-seq experiments. Compared to ENCODE and our previously published HeLa-S3 CTCF ChIP-seq data, our CTCF peaks overlapped well with published CTCF binding sites, indicating that CTCF ChIP-seq experiments produced good enrichment (Supplemental Figure S10A). We then compared CTCF ChIP-seq results between control shRNA and shRNA targeting BHLHE40. Interestingly, BHLHE40 loss-of-function led to a decreased number and enrichment of a subset of CTCF binding sites (Figure 5B and C). And this effect was not caused by a change in either the RNA or protein levels of CTCF (Supplemental Figure S10B and C), indicating that BHLHE40 might directly influence CTCF binding. To test if BHLHE40 forms a protein complex with CTCF, we performed co-immunoprecipitation (co-IP) experiment, and observed that FLAG-CTCF could precipitate BHLHE40 (Figure 5D), and we also constructed BIOTIN-tagged BHLHE40 HeLa-S3 stable cell line (Figure 5E) and BIOTIN immunoprecipitation results showed that BHLHE40 could also precipitate CTCF (Figure 5F), suggesting that BHLHE40 may modulate CTCF function. Meanwhile, we performed BHLHE40 ChIP-seq experiments, which yielded several hundred peaks, and all of these peaks were sensitive to BHLHE40 knockdown (lanes 2-5 of Figure 5G). To enhance the peak detection efficiency, we took advantage of biotin-tag system to investigate BHLHE40 binding (65). Using BIOTIN-tagged BHLHE40 stable cell lines, we performed BIOTIN ChIP-seq experiments for BHLHE40. Results showed that BIOTIN ChIP-seq data for BHLHE40 had a good enrichment (Lane 1 of Figure 5G), and the endogenous BHLHE40 also had a strong enrichment (lanes 2-3 of Figure 5G). Peak overlap analysis revealed that 2452 BHLHE40 peaks overlapped with CTCF binding sites (34.8%, 2452/7036) (lanes 1, 6, 7 of Figure 5G). Surprisingly, we found that the enrichment of CTCF at the overlapping regions was significantly decreased after BHLHE40 loss-of-function (lanes 8, 9 of Figure 5G). Validation of several loci by ChIP-qPCR confirmed a decreased CTCF enrichment following BHLHE40 knockdown (Figure 5H and Supplemental Figure S10D). However, the CTCF binding sites that overlapped with BHLHE40 peaks accounted for only a subset of the overall down-regulated CTCF binding sites, suggesting that BHLHE40 might regulate CTCF binding by other unknown mechanisms.

**Figure 6.**
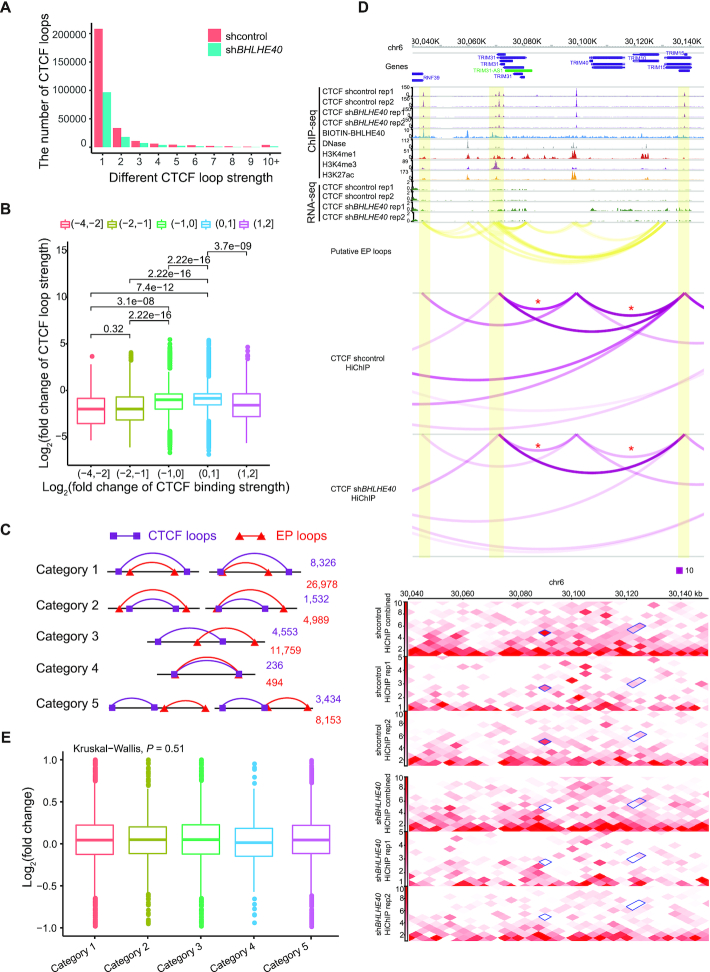
BHLHE40 depletion reduces CTCF-mediated chromatin loops. (**A**) Bar chart showing the number of CTCF loops with different loop strength in control shRNA and BHLHE40 shRNA-depleted HeLa-S3 cells. (**B**) Boxplot showing fold-change distribution of loop strength in different groups of CTCF loop anchors, which are classified by the fold change of CTCF binding strength between control shRNA and BHLHE40 shRNA-depleted HeLa-S3 cells. *P* value was calculated using Wilcoxon rank sum test. (**C**) The position relationship between differential CTCF loops resulted by BHLHE40 depletion and putative EP loops. Category 1 represents CTCF loops containing EP loops. Category 2 represents EP loops containing CTCF loops. Category 3 represents CTCF loops intersecting with EP loops. Category 4 represents that CTCF loops are the same as EP loops. Category 5 represents that CTCF loops do not intersect with EP loops. (**D**) Screenshot from the WashU epigenome browser showing the change of CTCF loops between control shRNA and BHLHE40 shRNA-depleted HeLa-S3 cells. The tracks of DNase, H3K4me1, H3K4me3 and H3K27ac ChIP-seq data were downloaded from the Roadmap Epigenome project (50). Chromatin interaction heatmaps were shown in 5 kb resolution. Significantly differential loops were marked with red asterisk. (**E**) Distribution of the expression fold change in different groups of genes classified by the position relationship between CTCF loops and EP loops. *P* value was calculated using Kruskal-Wallis test.

The reduction of CTCF binding by loss of BHLHE40 might lead to changes in CTCF loops. To examine this, we performed CTCF HiChIP experiments in both control shRNA and BHLHE40-depleted cells. CTCF HiChIP data in control cells were first compared to our previously published HiChIP data, the results indicated that they were well correlated (Supplemental Figure S11A and B). Furthermore, our CTCF HiChIP results also correlated well with each other and had very similar chromatin loops in two different replicates (Supplemental Figure S11C). Therefore, we combined and analyzed CTCF HiChIP data together for further study. Our results showed that BHLHE40 loss-of-function reduced CTCF loop strength (Figure 6A and Supplemental Figure S11D), which might be caused by reduced CTCF binding (Figure 6B). Further, we explored the consequences of reduced CTCF loops caused by BHLHE40 depletion. CTCF loops maintain genome structures and participate in regulating gene expression by disrupting enhancer-promoter loops (EP loops) (66,67). In order to investigate the relationship between reduced CTCF loops and EP loops, we generated putative EP loops (see Materials and Methods), and categorized the EP loops and decreased CTCF loops based on the overlap relationship of loop anchor. We classified these loops into five categories, as depicted in Figure 6C. Each EP loop or CTCF loop might be involved in multiple categories. In specific gene loci, we found that a gene could have multiple EP loops and nearby CTCF loops, resulting in a very complicated loop relationship (Figure 6D and Supplemental Figure S12). Usually, the pattern of CTCF-mediated loops influencing gene expression is to interfere with EP loops which is illustrated as category 3 (Figure 6C). We found that a small portion of differential CTCF loops intersected with putative EP loops, and the majority of the remaining CTCF loops contained or were contained in EP loops (Figure 6C). To further explore whether the expression of genes involved in category 3 was changed after BHLHE40 depletion, RNA-seq was performed. However, down-regulation of BHLHE40 seemed to have little effect on gene expression in HeLa-S3 cells (Supplemental Figure S11E and F). For some genes, the decreased CTCF loops may lead to the enhancement of EP loop interactions, which causes the increase of gene expression (Figure 6D). For all genes involved in category 3, the impact of decreased CTCF loops on gene expression seems limited (Figure 6E). It seems that the genes have multiple enhancers, and the enhancement of several EP loops caused by the decreased CTCF loops could not significantly influence gene expression (Supplemental Figure S12). These data suggest that BHLHE40 depletion might result in the reduction of CTCF loops but have little effect on gene expression in HeLa-S3 cells (Figure 6E).

## DISCUSSION

The DNA sequences of the eukaryotic genome form a complex three-dimensional architecture, and CTCF serves as a chromatin looping mediator to build the higher-order genome structure (68,69). Identification of CTCF-associated co-factors may help us to understand the varied genome structure in different cell types. In this paper, we exploited the cell type-independent pattern of CTCF binding to define potential co-factor binding proteins. This study suggested that, compared with general genome-wide CTCF binding sites, using hscCTCF binding sites might be more convenient to compare the overlap ratio between CTCF and CTCF co-binding factors. Therefore, taking advantage of hscCTCF binding sites could provide a new method to identify CTCF co-binding factors.

In addition to previously reported co-factors, such as RAD21 and SMARCA4, we identified many new factors that could overlap strongly with CTCF, and these factors provide a new opportunity to study the mechanism of CTCF loop dynamics. Among these factors, we found that the overlap ratio of cohesin subunits in different cells was relatively high and stable, but the overlap ratio of many other factors fluctuated in different cell types. This might be due to the different ChIP enrichment for each factor or CTCF, which resulted in different peak number and caused variation of the overlap ratio. Except for ChIP-seq data quality, context-dependent factor binding would also lead to large differences in CTCF co-localization. On the one hand, although there are many conserved CTCF binding sites, the cell-specific CTCF binding sites still have different fractions in different cell types (59,70). On the other hand, the expression levels and binding pattern of many transcription factors in different cells are quite different (71). We selected ChIP-seq datasets for all factors in GM12878, HeLa-S3, K562, MCF7 cell lines and performed overlap analysis with CTCF binding sites. We found that it is difficult to compare the overlap ratio in different cell types (Supplemental Figure S13A). When performing overlap analysis with hscCTCF binding sites (Supplemental Figure S13B), the overlap ratio of many factors with hscCTCF binding sites is much higher than that of these factors with general CTCF binding sites, such as RAD21, SMC3, suggesting the results of overlap analysis with hscCTCF might be much better.

In addition to co-localization analysis, we explored information related to CTCF loops in four specific cell types. We found that factors clustered with CTCF in the loop anchor regions, and the anchor with more factors had a greater capacity to mediate stronger loops. And we also observed that many CTCF binding sites were located at gene promoter and enhancer regions with strong histone modifications, where chromatin loops were usually mediated by mediator complex. CTCF could mediate long-range chromatin interactions, and have long been known to have enhancer-blocking function to inactivate important genes (72–75). Different from this canonical function, several reports have been shown that CTCF-mediated chromatin loops could be also involved in EP loops (76–79). By investigating CTCF function in promoter and enhancer regions, our results revealed that CTCF binding strength in promoter and enhancer regions had good correlation with the EP loop strength (Supplemental Figure 14), suggesting that CTCF might directly participate in and facilitate EP loops. The two-side effects on gene expression regulation which mediated by CTCF loops increased the complexity of CTCF function, and systematic proofs to identify which genes were regulated by this pattern still need to be further investigated in detail.

Overall, our study used the unique properties of CTCF to computationally predict co-factors for CTCF. We went on to experimentally validate BHLHE40 as a co-factor of CTCF, and showed that it did co-localize with CTCF and knockdown of BHLHE40 led to a reduction in CTCF binding, and a reduction in CTCF-mediated loop strength. Nonetheless, our identified co-factors for CTCF may both positively and negatively regulate CTCF-mediated functions and future research will reveal the roles of these multi-component CTCF complexes.

## DATA AVAILABILITY

Our sequencing data for ChIP-seq, RNA-seq and HiChIP have been deposited in the Gene Expression Omnibus and the accession numbers are: GSE137848, GSE137849, GSE137850, respectively. All other relevant data supporting the key findings of this study are available within the article and supplementary information files.

## Supplementary Material

gkaa705_Supplemental_Files

## Notes

Present address: Key Laboratory of Regenerative Biology, South China Institute of Stem Cell & Regenerative Medicine, Guangzhou Institutes of Biomedicine and Health, Chinese Academy of Sciences. 190 Kai Yuan Avenue, Science Park Guangzhou 510530, China.
