# Supplementary material for: Systematic screening of CTCF binding partners identifies that BHLHE40 regulates CTCF genome-wide distribution and long-range chromatin interactions
Source: Nucleic Acids Res. 2020 Sep 4;48(17):9606–20. doi: 10.1093/nar/gkaa705 (PMC7515718; doi:10.1093/nar/gkaa705)

Supplementary information

Supplemental information includes 14 supplemental figures and 7 supplemental tables.

Supplementary Figure Legends

Supplemental Figure S1. Chromatin interaction mediated by CTCF in different cell lines, respectively. (**A**) Chromatin interaction heatmaps in partial region of human chromosome 7. (**B**) Tracks showing the chromatin interactions mediated by CTCF binding sites. The yellow rectangle highlights the conserved CTCF binding sites. (**C**) Bar plot showing the number of CTCF binding sites in 23 cell lines.

Supplemental Figure S2. CTCF binding strength between hscCTCF binding sites and all CTCF binding sites in (**A**) GM12878 cells, (**B**) HeLa-S3 cells, (**C**) K562 cells and (**D**) MCF7 cells, respectively.

Supplemental Figure S3. Co-localization heatmap between each protein co-factor binding sites with CTCF binding sites in four cell types. 2 kb regions flanking the summits of CTCF binding sites were used.

Supplemental Figure S4. The relationship between CTCF co-localization factors and CTCF-mediated chromatin interactions among different groups of CTCF binding sites. (**A**) Heatmaps showing CTCF co-localized factors in three different cell types grouped by hierarchical clustering. (**B**) Violin plots showing the distribution of CTCF peak signal which is the value of seventh column in the narrowPeak file generated by MACS2 among 4 groups of

CTCF binding sites. (**C**) Violin plots showing CTCF loop strength distribution among 4 groups of CTCF binding sites. For each loop anchor, loop strength was calculated by summing up all loops counts mediated by the anchor. Y axis is $\log_{10}$ scaled.

Supplemental Figure S5. The relationship between each protein factor and CTCF loops in HeLa-S3 cells. (**A**) Heatmap showing $-\log_{10}$(FDR) of CTCF loop strength between factor cobinding sites and factor non-cobinding sites. *P* value is calculated with Wilcoxon rank sum test and further adjusted for multiple comparisons using Bonferroni correction. (**B**) Heatmap showing the correlation between factor binding strength and CTCF loops. Factors with maximum correlation more than 0.2 are selected. (**C**) Network visualization displaying the frequency of factor appearance in paired loop anchors.

Supplemental Figure S6. The relationship between each protein factor with CTCF loops in K562 cells. (**A**) Heatmap showing $-\log_{10}$(FDR) of CTCF loop strength between factor cobinding sites and factor non-cobinding sites. *P* value is calculated with Wilcoxon rank sum test and further adjusted for multiple comparisons using Bonferroni correction. (**B**) Heatmap showing the correlation between factor binding strength and CTCF loops. Factors with maximum correlation more than 0.2 are selected. (**C**) Network visualization displaying the frequency of factor appearance in paired loop anchors.

Supplemental Figure S7. The relationship between each protein factor with CTCF loops in MCF7 cells. (**A**) Heatmap showing $-\log_{10}$(FDR) of CTCF loop strength between factor cobinding sites and factor non-cobinding sites. *P* value is calculated with Wilcoxon rank sum test and further adjusted for multiple comparisons using Bonferroni correction. (**B**) Heatmap

showing the correlation between factor binding strength and CTCF loops. Factors with maximum correlation more than 0.1 are selected. (**C**) Network visualization displaying the frequency of factor appearance in paired loop anchors.

Supplemental Figure S8. The correlation between binding strength of either RAD21 or CTCF with CTCF-mediated loop strength. The scatter plots showing the correlation between (**A**) RAD21 binding strength, (**B**) CTCF binding strength with $\log_2$ transformed CTCF loop strength in four cell types.

Supplemental Figure S9. The annotation of CTCF binding sites in different chromatin elements. Regions with similar function were classified together. TssA, TssFlnk, TssFlnkU and TssFlnkD regions were grouped into promoter regions. Tx and TxWk regions were grouped into transcription regions. EnhG1, EnhG2, EnhA1, EnhA2 and EnhWk regions were grouped into enhancer regions.

Supplemental Figure S10. Experimental exploration of BHLHE40 on CTCF binding and its mediated chromatin interactions. (A) Venn plot showing CTCF peak overlap analysis among HeLa-S3 control ,our previously published data and ENCODE. (B) Bar plot showing CTCF expression after BHLHE40 knock-down. (**C**) Western blot showing CTCF protein level after BHLHE40 knock-down. (**D**) WashU epigenome browser views of one representative BHLHE40/CTCF common binding site. ChIP-qPCR results showing the decrease of BHLHE40 and CTCF enrichment following BHLHE40 knock-down. Data in (B) and (D) are from three biological replicates and represented as mean± SEM. *$P < 0.05$. P value is calculated using two-tailed Student's *t* test.

Supplemental Figure S11. The effect of BHLHE40 depletion on CTCF-mediated chromatin loops and gene expression. (A) Correlation analysis between CTCF HiChIP data in control shRNA HeLa-S3 cells and our previously published CTCF HiChIP data. (B) Correlation analysis of CTCF HiChIP data between control shRNA and BHLHE40-depleted HeLa-S3 cells. (C) WashU epigenome browser view of CTCF-mediated chromatin loops from CTCF HiChIP data with two replicates. (D) WashU epigenome browser view of CTCF, BHLHE40 ChIP-seq data and CTCF-mediated chromatin loops in control shRNA and BHLHE40-depleted HeLa-S3 cells. (E) BHLHE40 gene expression assessed by the count number of RNA-seq data in control shRNA and BHLHE40-depleted HeLa-S3 cells. (**F**) The number of significantly differential expressed genes between control shRNA and BHLHE40-depleted HeLa-S3 cells.

Supplemental Figure S12. WashU epigenome browser views of ChIP-seq, RNA-seq, CTCF HiChIP and putative enhancer-promoter loops in control shRNA and BHLHE40 HeLa-S3 cells. The tracks of DNase, H3K4me1, H3K4me3 and H3K27ac ChIP-seq data were download from the roadmap epigenome project (49). Chromatin interactions in heatmaps were shown in 5 kb resolution for Figure A and 10 kb resolution for Figure B. Significantly differential loops were marked with red asterisk.

Supplemental Figure S13. Overlap analysis between CTCF binding sites and factor binding sites in GM12878, HeLa-S3, K562 and MCF7 cells. (**A**) Scatter plot showing the top 50 factors overlapped with all CTCF binding sites ranked by maximum overlap ratio. (**B**) Scatter plot showing the top 50 factors overlapped with hscCTCF binding sites ranked by maximum overlap ratio.

Supplemental Figure S14. The correlation analysis between the normalized CTCF binding strength and log_2 transformed CTCF loop strength in the (**A**) promoter and (**B**) enhancer regions in GM12878, HeLa-S3 and K562 cells.

Supplemental Tables

Supplemental Table S1. Sequence lists for RT-qPCR primers, ChIP-qPCR primers and shRNA oligos.

Supplemental Table S2. The information of hscCTCF binding sites.

Supplemental Table S3. Datasets from all transcription factors used for performing overlap analysis with corresponding CTCF binding sites in each cell type.

Supplemental Table S4. Datasets from all transcription factors used for performing overlap analysis with hscCTCF binding sites.

Supplemental Table S5. Maximum overlap ratio for each factor with hscCTCF binding sites.

Supplemental Table S6. The information of transcription factors used in Figure 3A and Supplemental Figure S4A.

Supplemental Table S7. DiffBind results of CTCF ChIP-seq in control shRNA and BHLHE40-depleted HeLa-S3 cells

Supplemental Table S8. Significantly up/down-regulated genes in BHLHE40-depleted HeLa-S3 cells compared with control shRNA HeLa-S3 cells.

Supplemental Table S9. CTCF loops identified from CTCF HiChIP data in control shRNA and BHLHE40-depleted HeLa-S3 cells. CTCF binding sites overlapped with each anchor were added in this table. NA represents no CTCF site, multiple CTCF sites in one anchor region are connected with commas.

Supplemental Figure S1

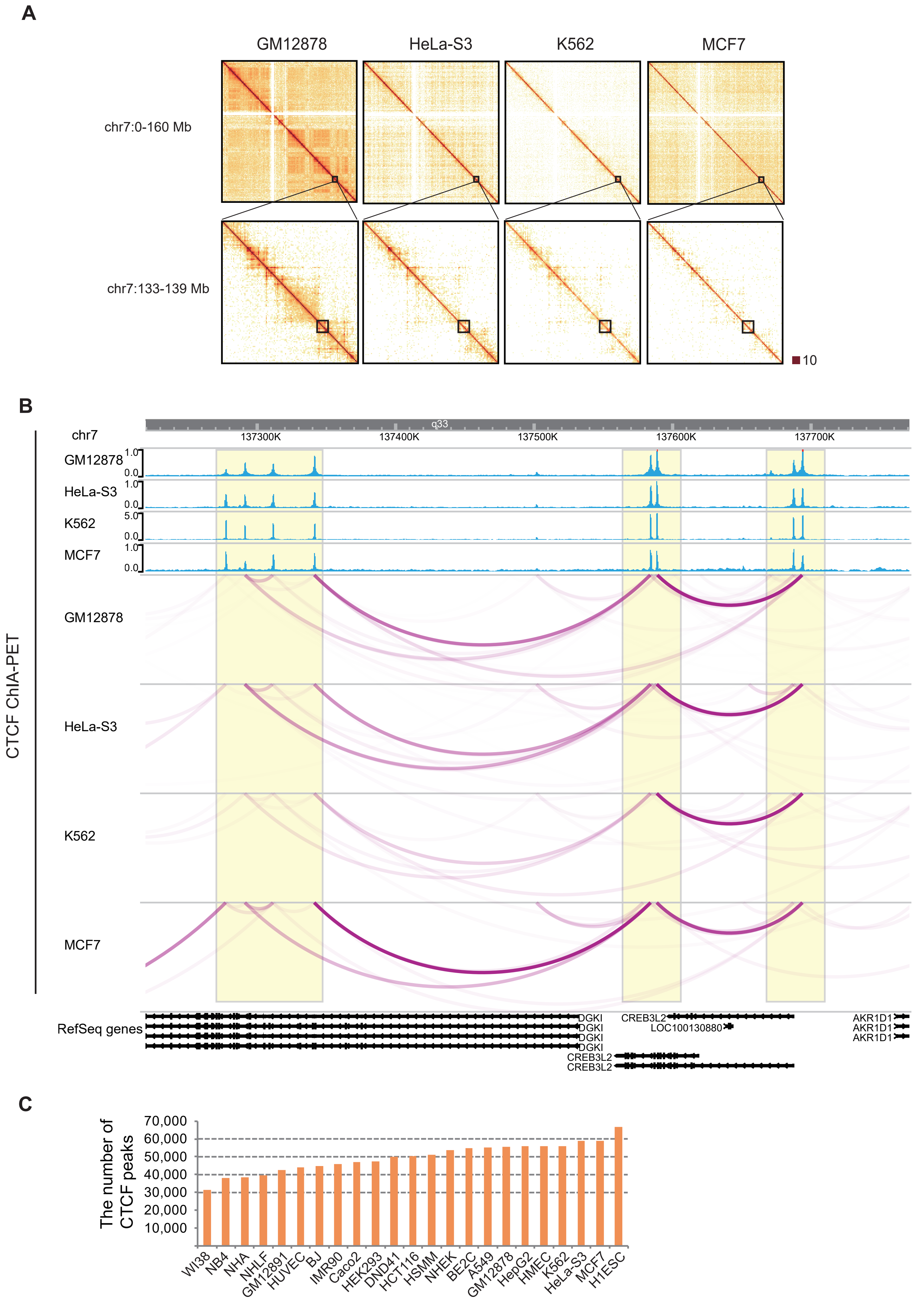

Supplemental Figure S2

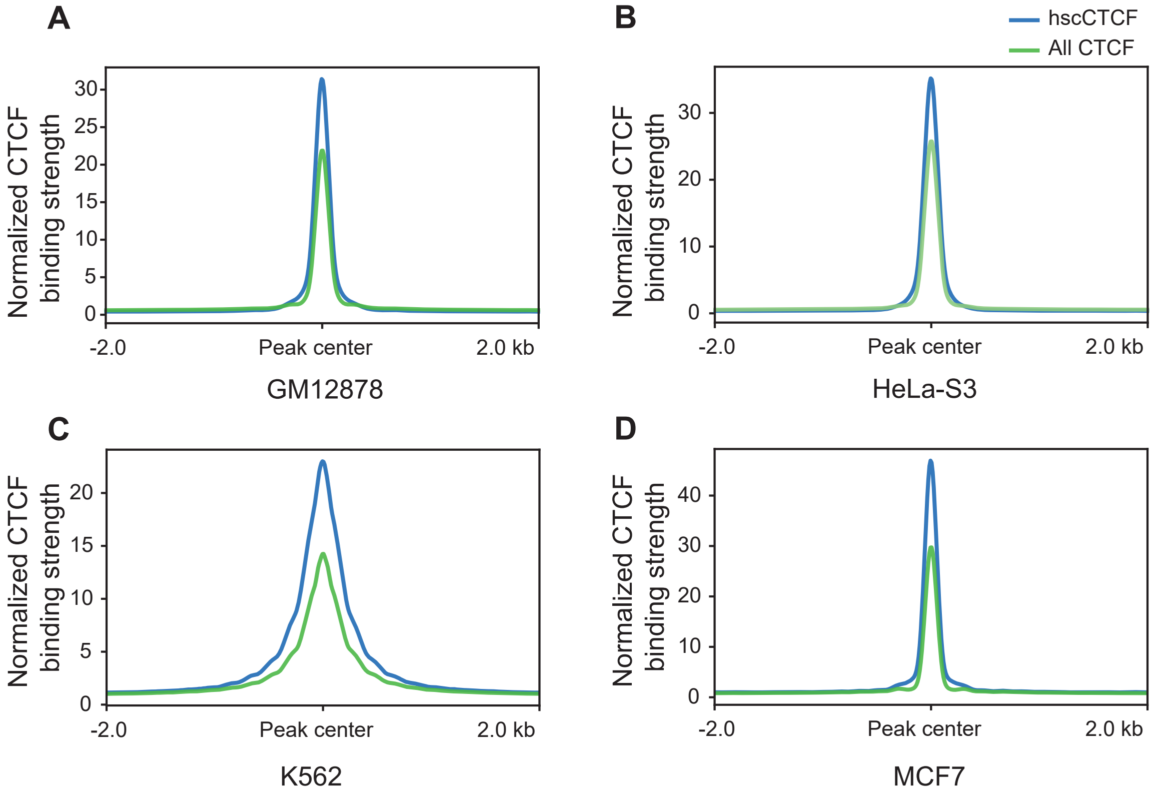

Supplemental Figure S3

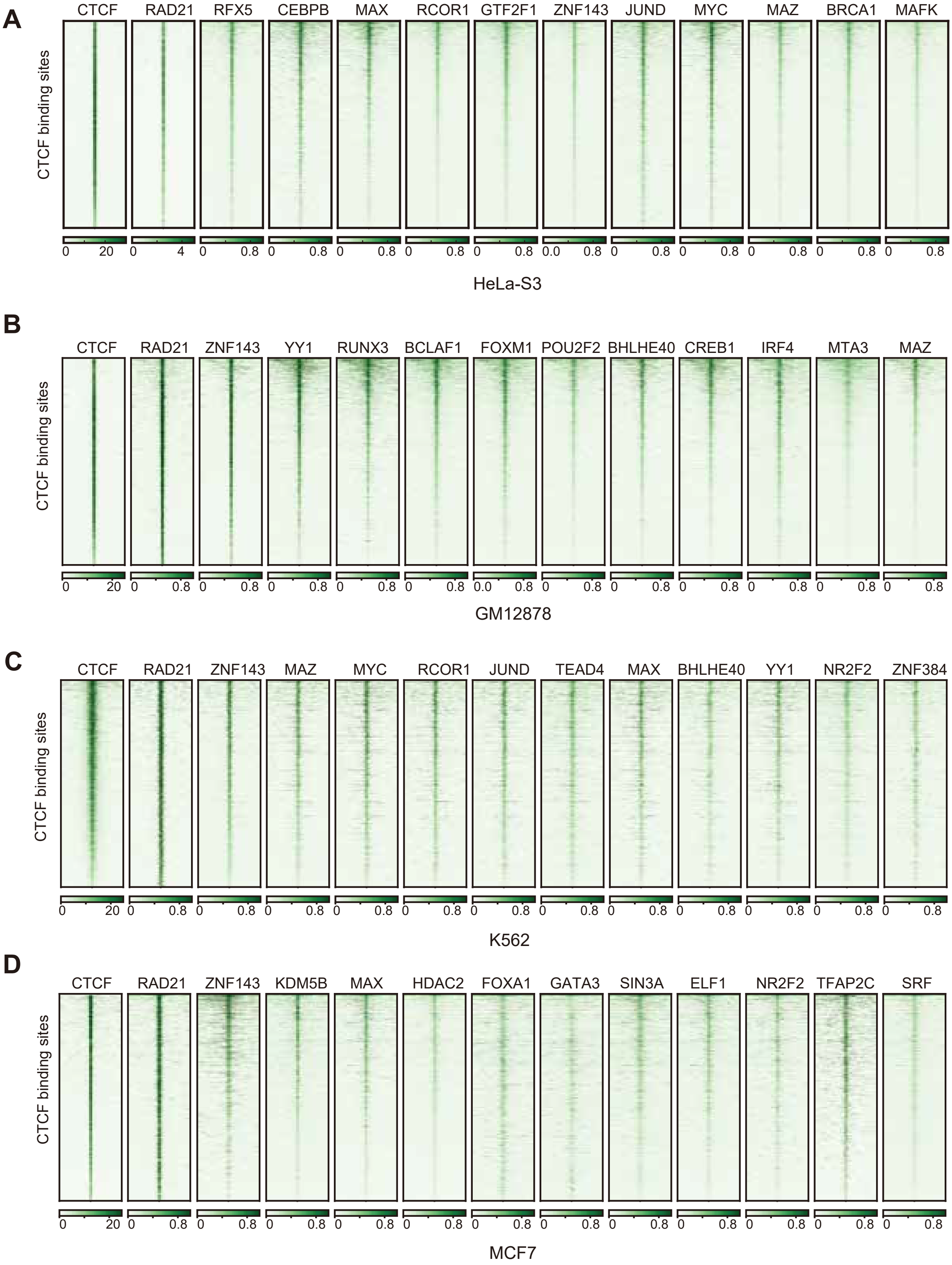

Supplemental Figure S4

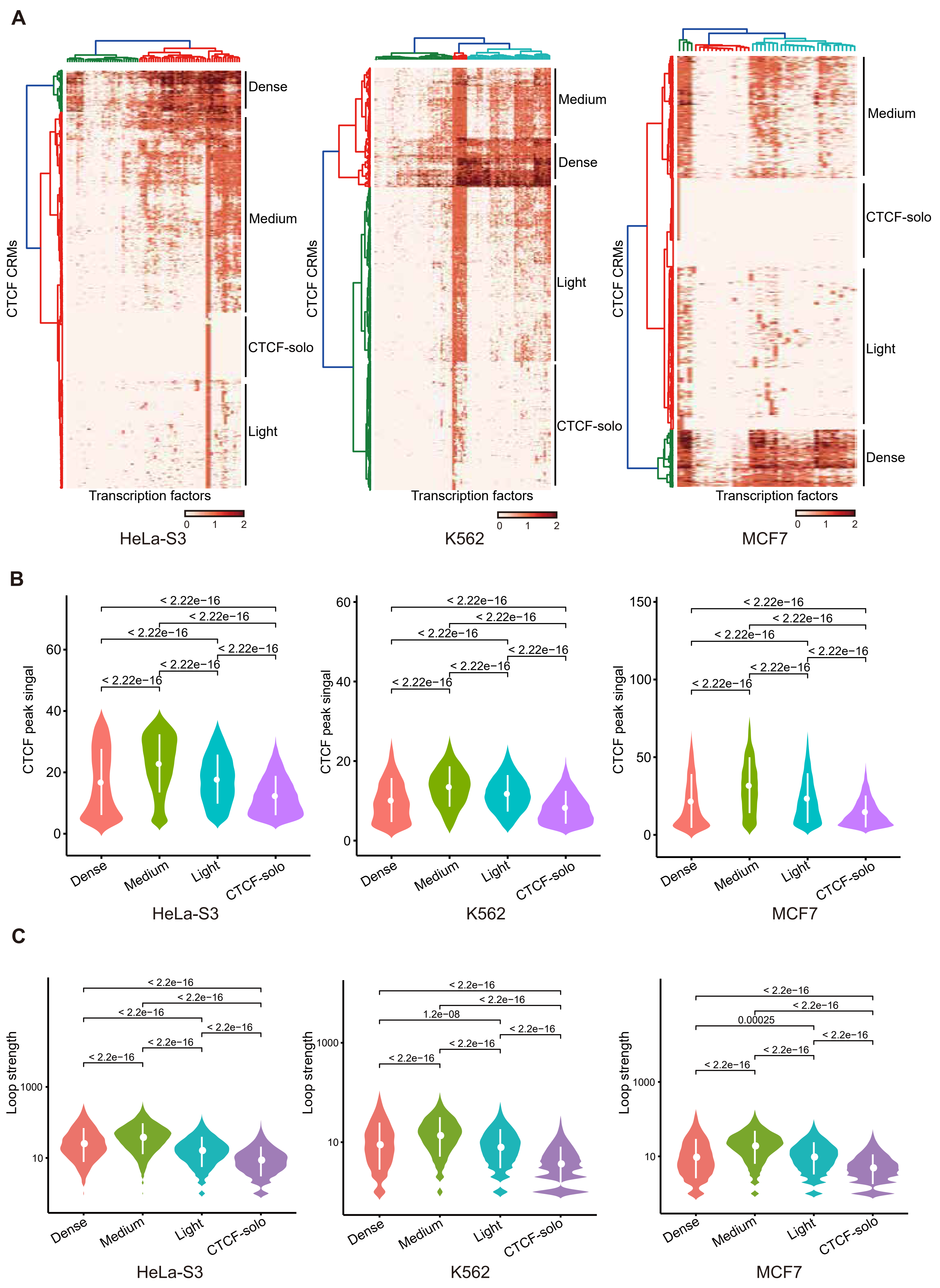

Supplemental Figure S5

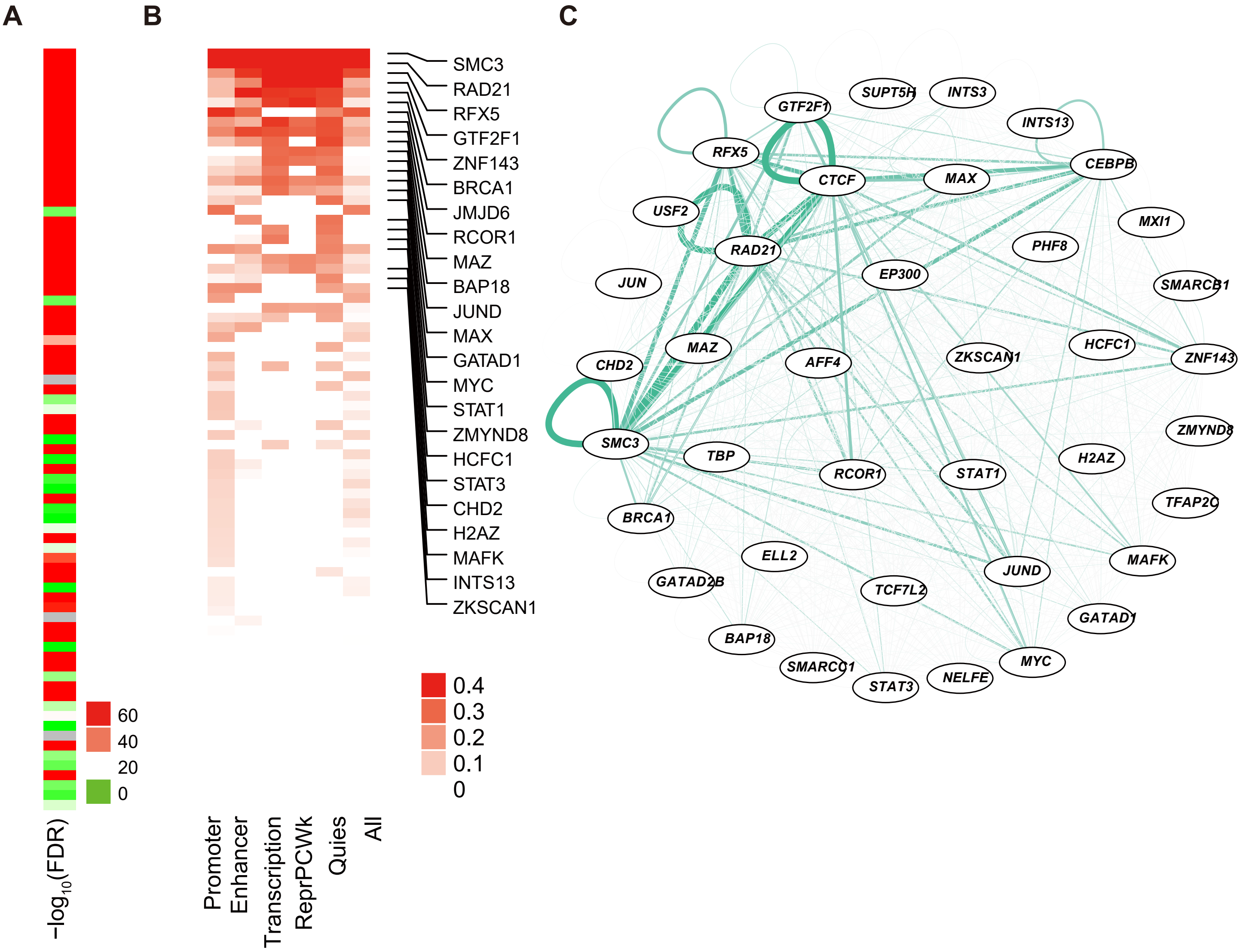

Supplemental Figure S6

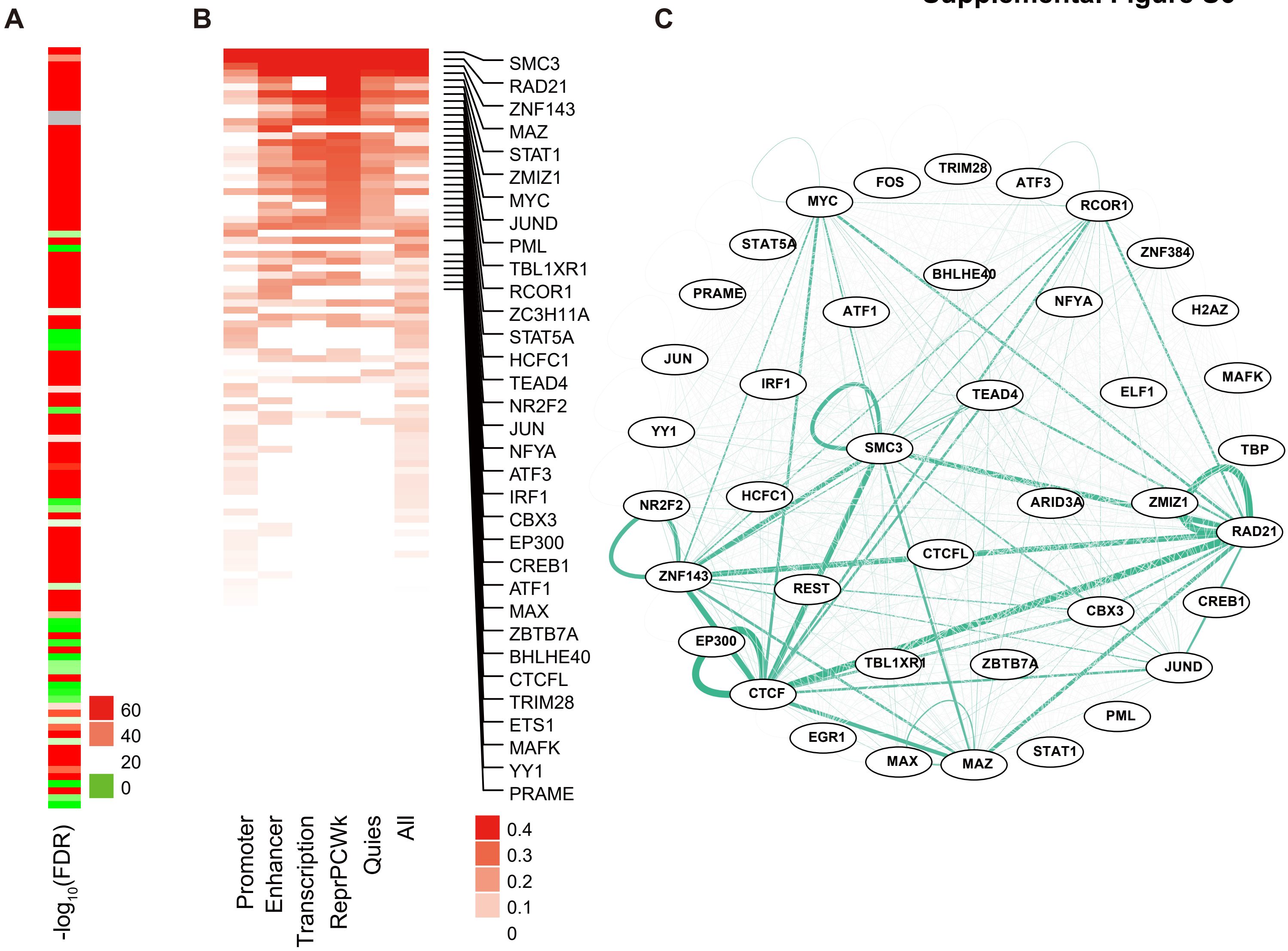

Supplemental Figure S7

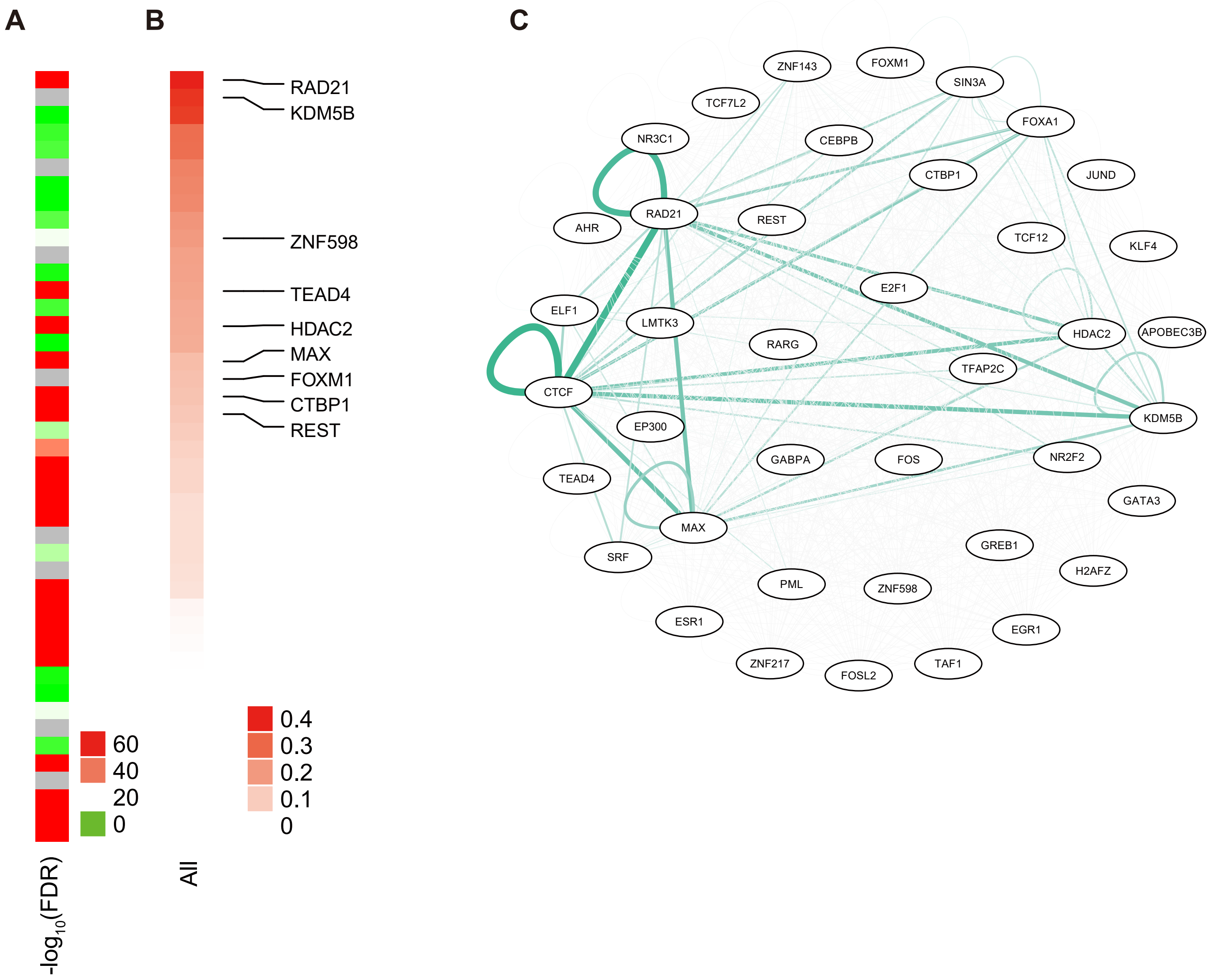

Supplemental Figure S8

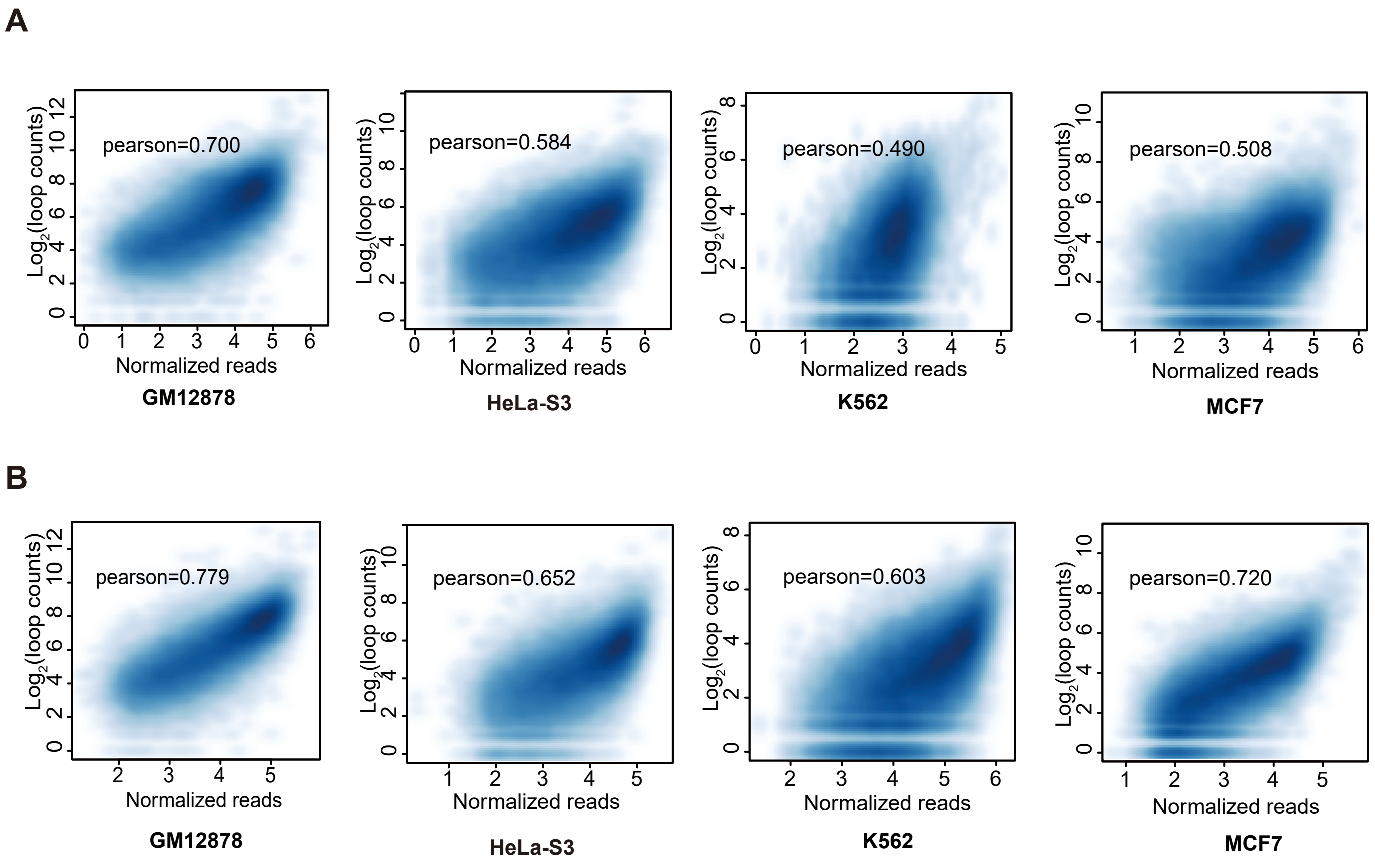

Supplemental Figure S9

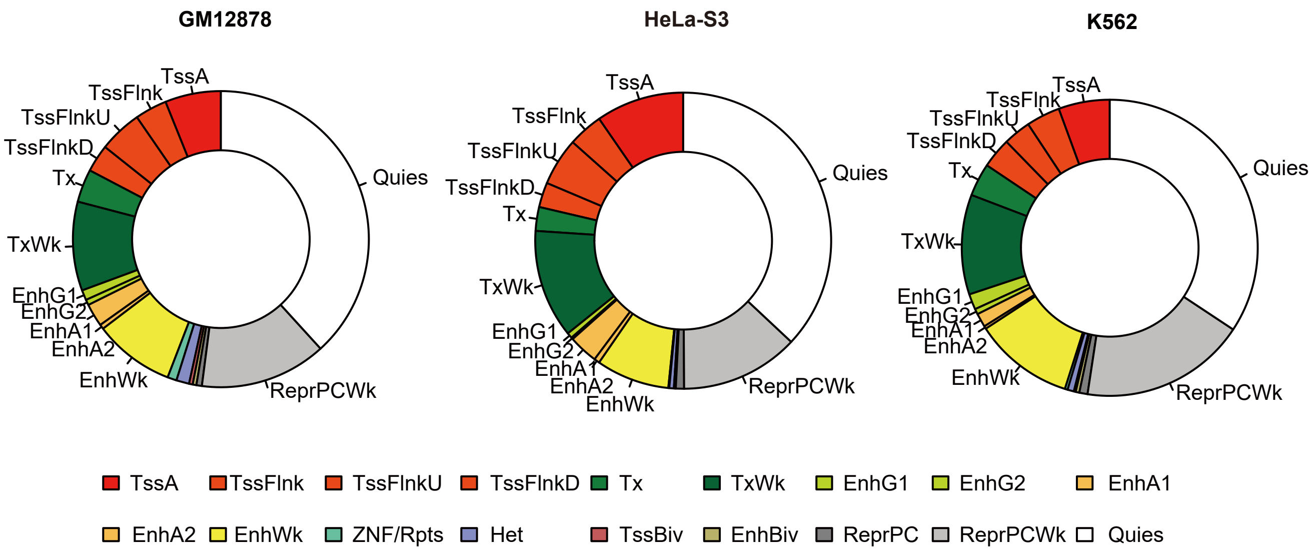

Supplemental Figure S10

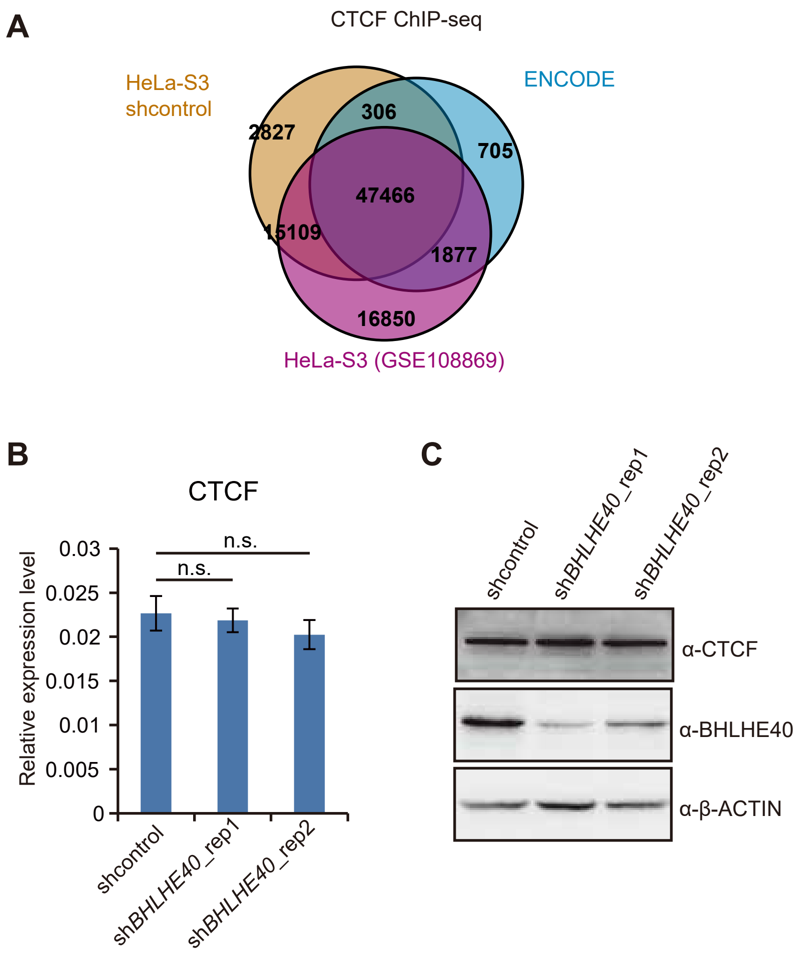

Supplemental Figure S11

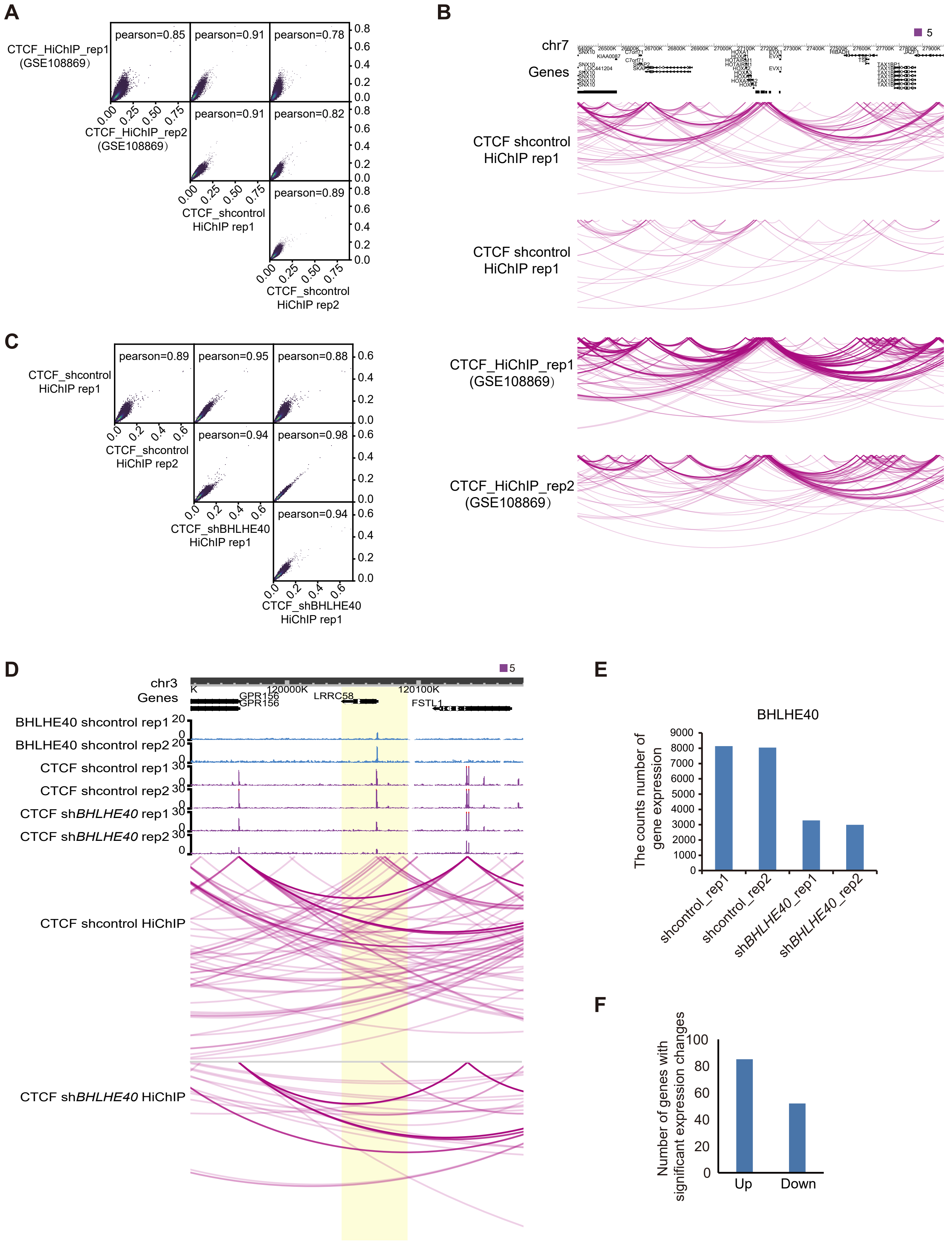

Supplemental Figure S12

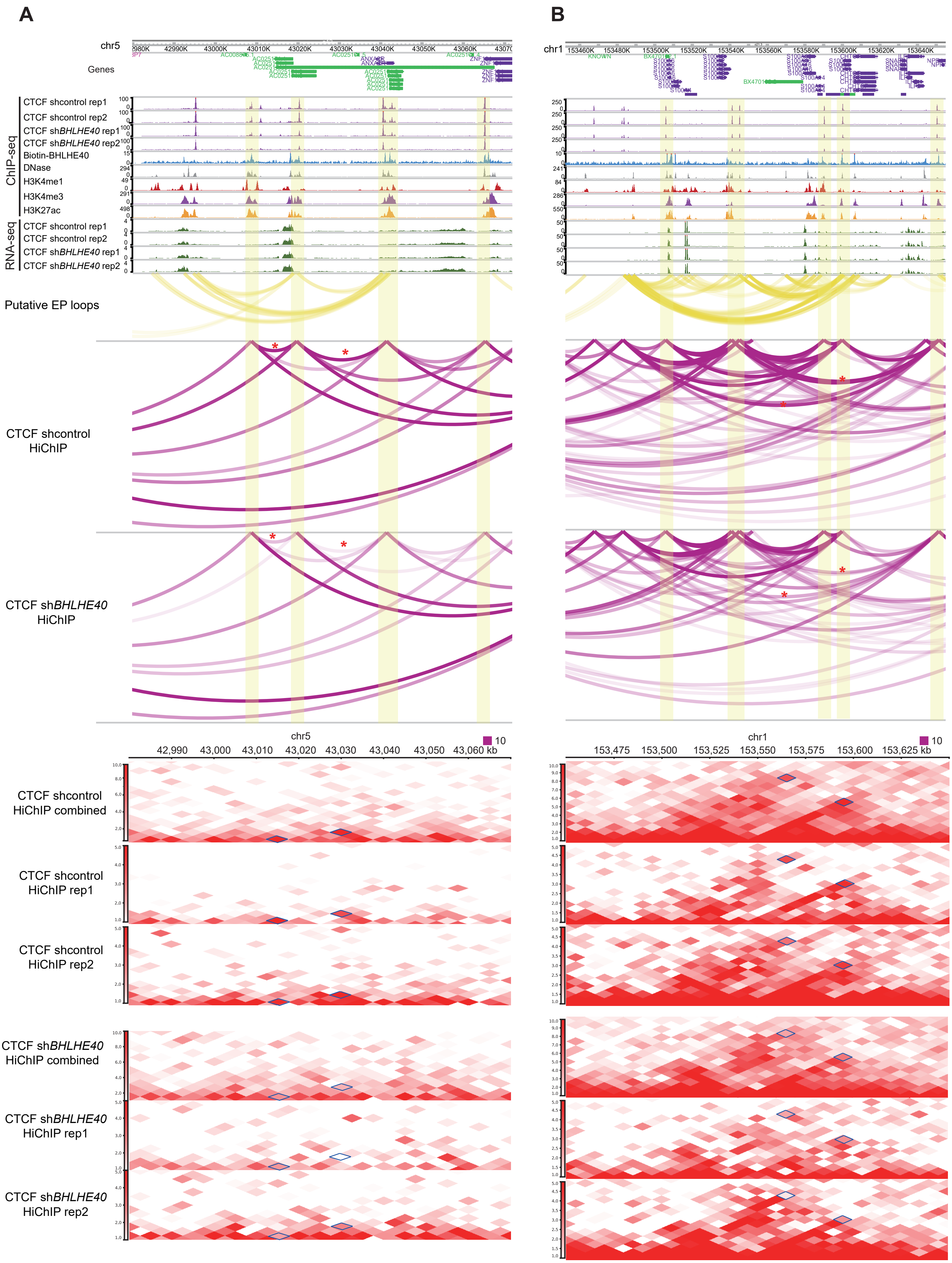

Supplemental Figure S13

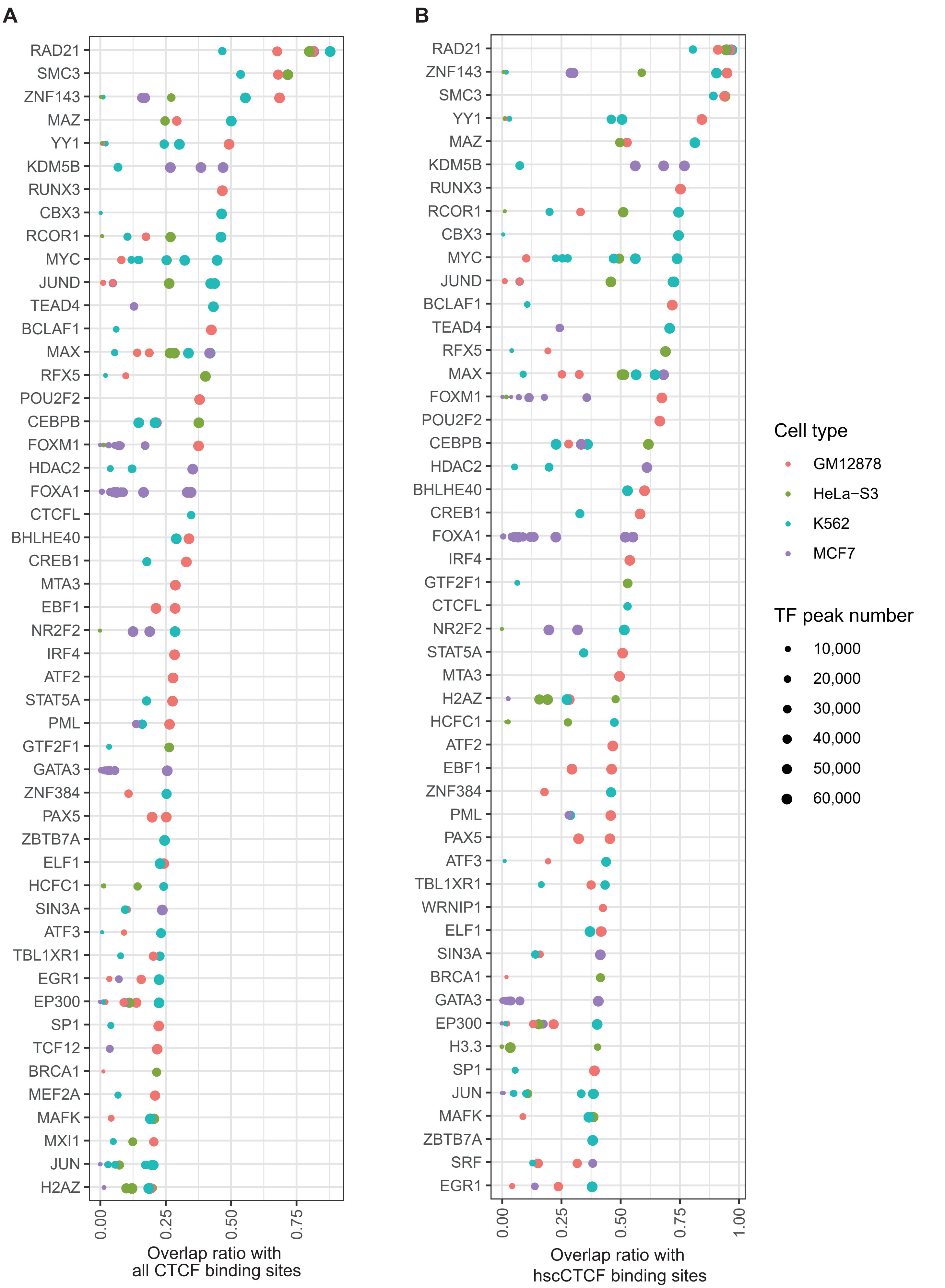

Supplemental Figure S14

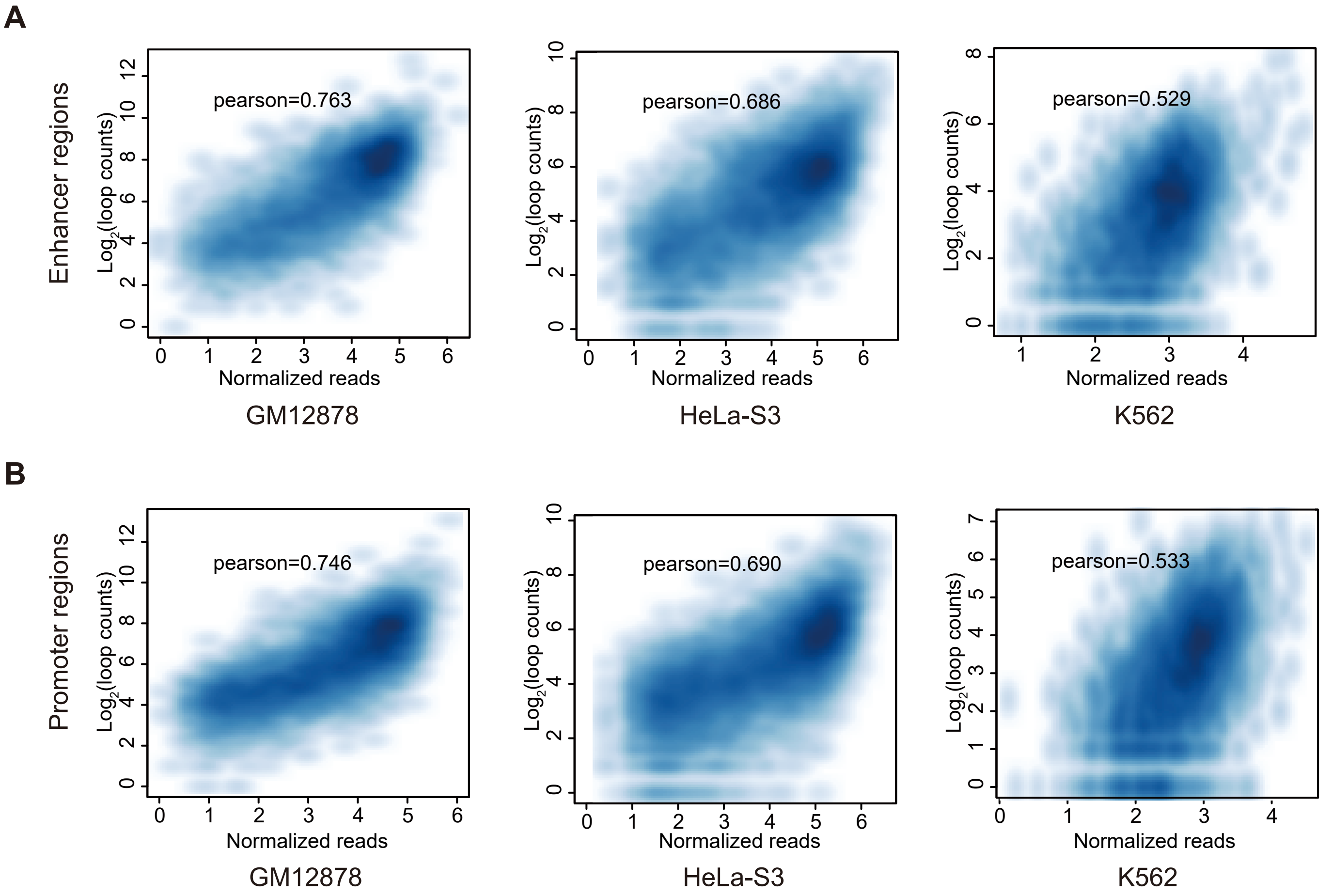

Supplement: gkaa705_Supplemental_Files [file gkaa705_supplemental_files.zip › Supplemental_Material_combined.pdf]
