# Supplementary figures and images for: Systematic screening of CTCF binding partners identifies that BHLHE40 regulates CTCF genome-wide distribution and long-range chromatin interactions
Source: Nucleic Acids Res. 2020 Sep 4;48(17):9606–20. doi: 10.1093/nar/gkaa705 (PMC7515718; doi:10.1093/nar/gkaa705)

Figure 1

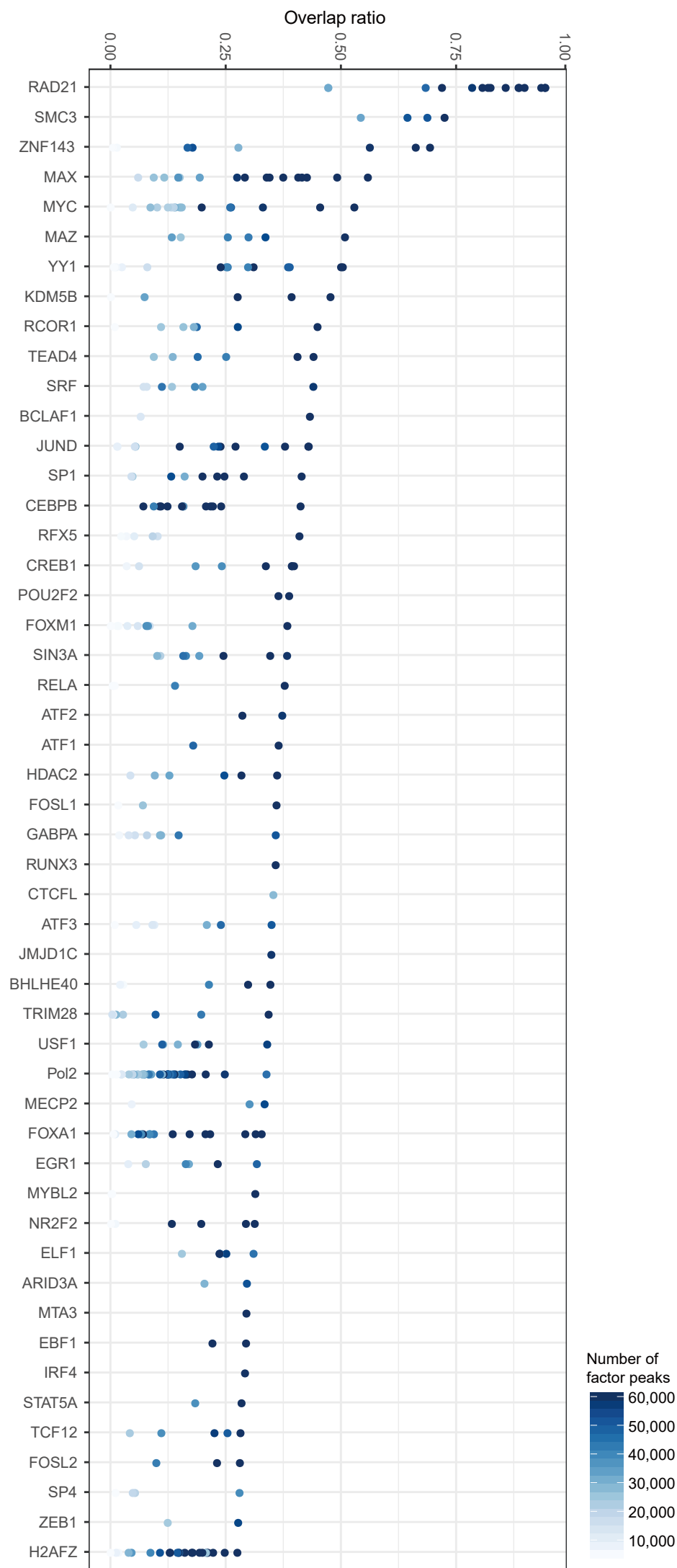

Supplement: gkaa705_Supplemental_Files [file gkaa705_supplemental_files.zip › Gongcheng_Fig1.pdf]

Figure 2

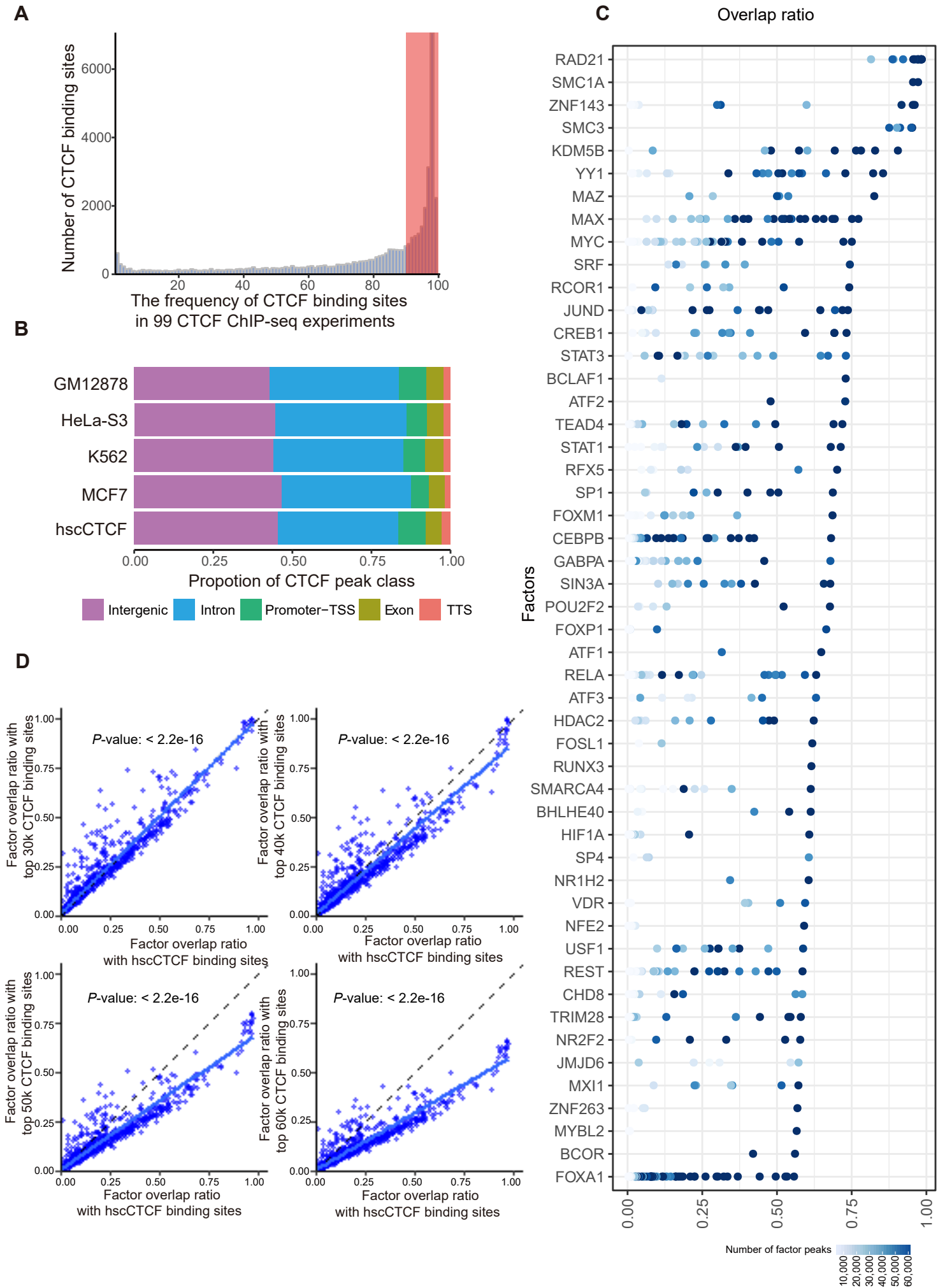

Supplement: gkaa705_Supplemental_Files [file gkaa705_supplemental_files.zip › Gongcheng_Fig2.pdf]

Figure 3

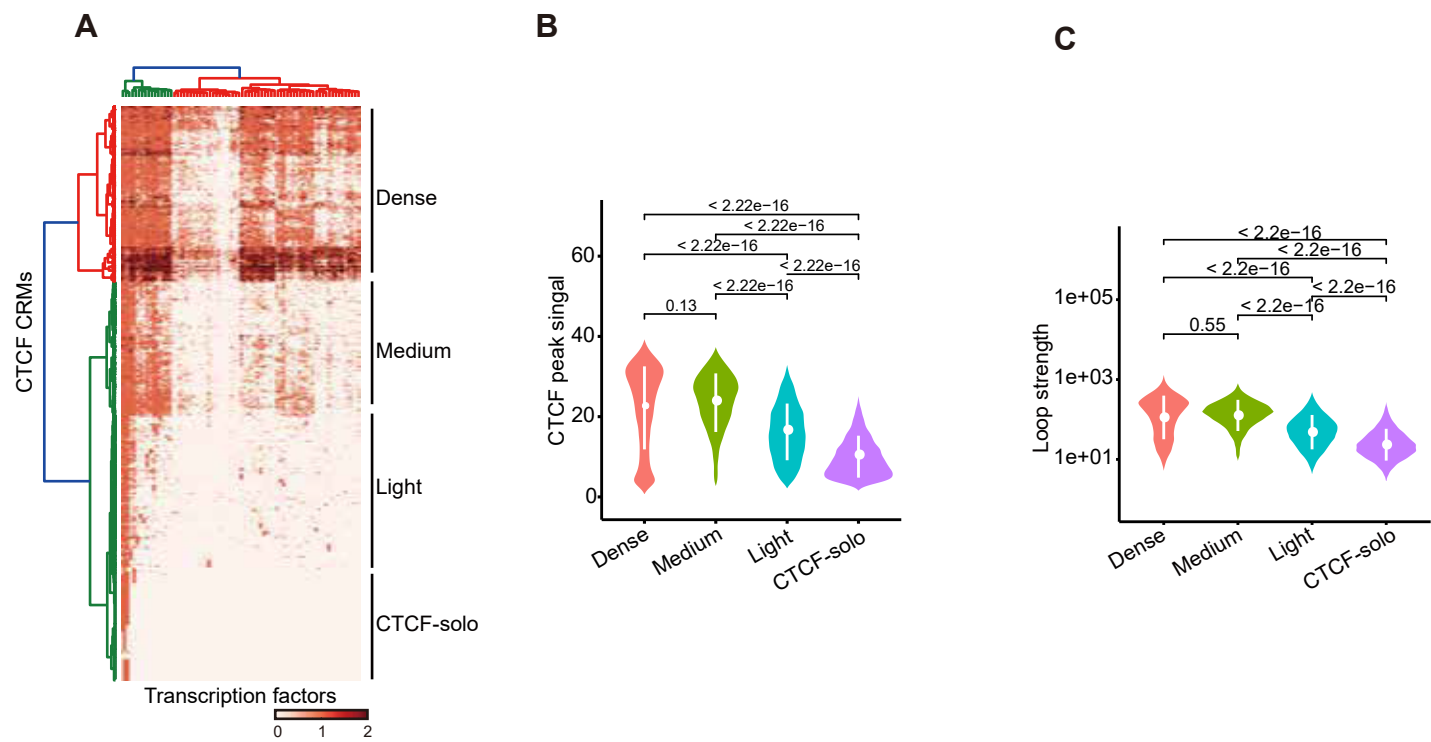

Supplement: gkaa705_Supplemental_Files [file gkaa705_supplemental_files.zip › Gongcheng_Fig3.pdf]

Figure 4

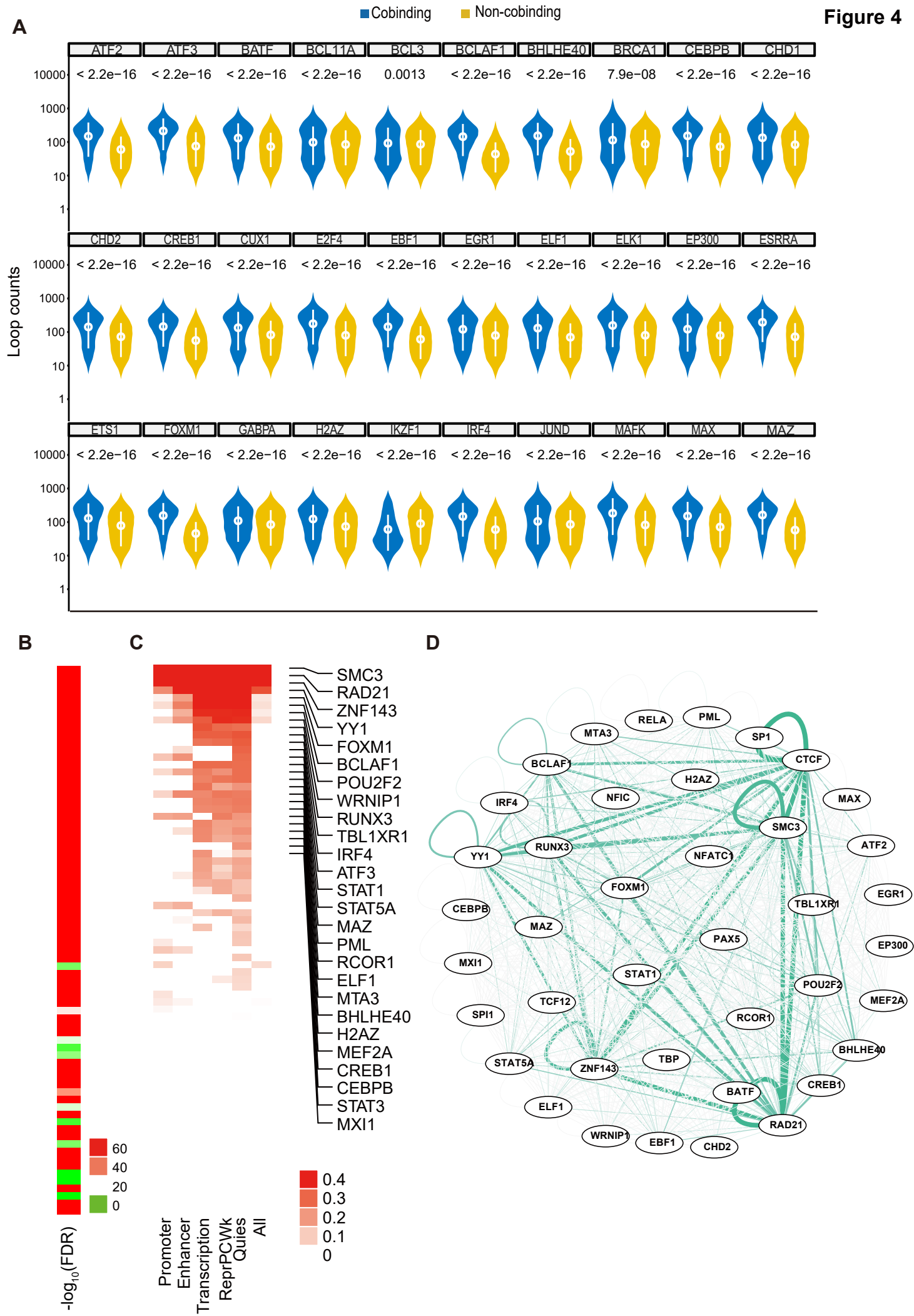

Supplement: gkaa705_Supplemental_Files [file gkaa705_supplemental_files.zip › Gongcheng_Fig4.pdf]

**Figure 5**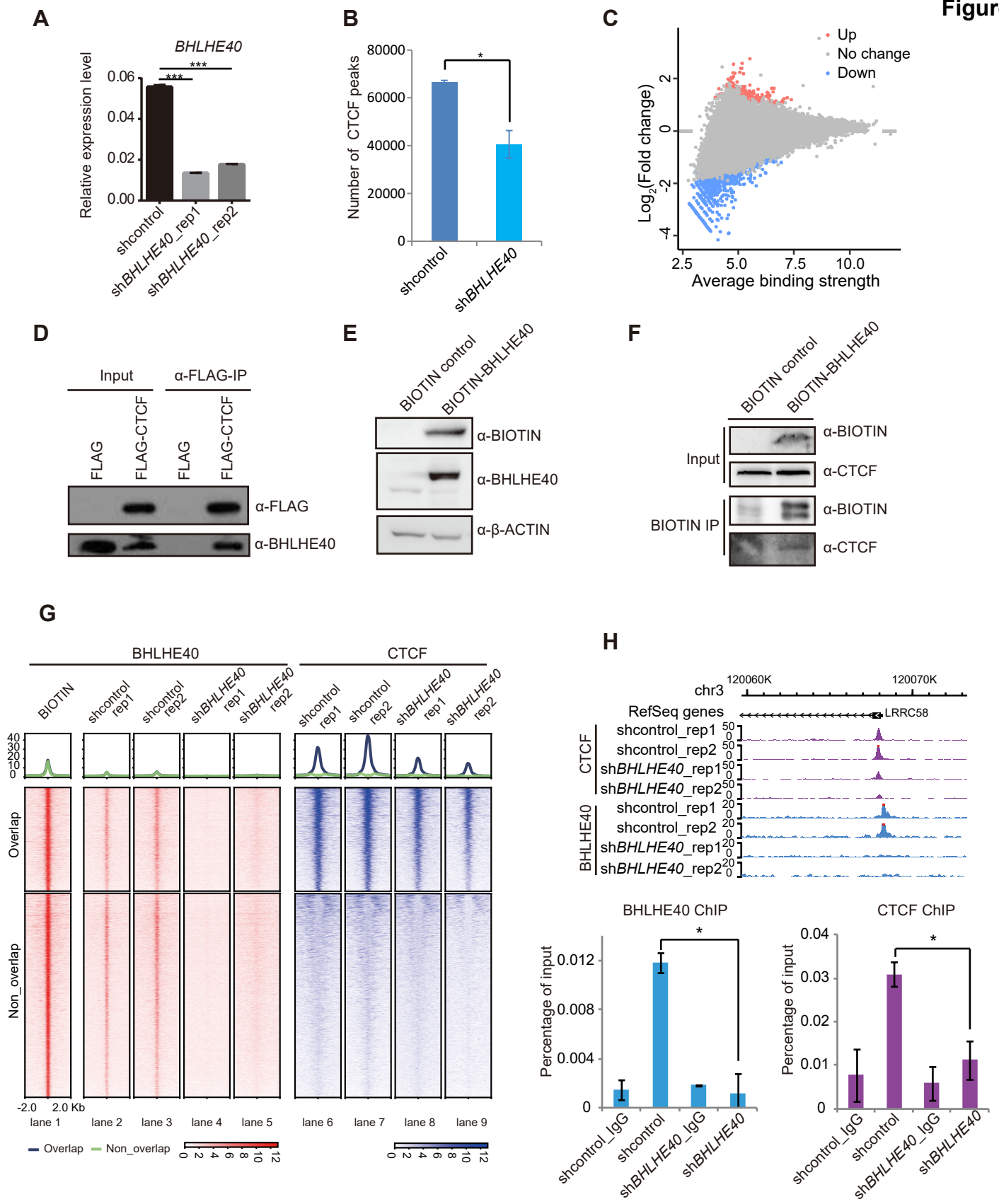

Supplement: gkaa705_Supplemental_Files [file gkaa705_supplemental_files.zip › Gongcheng_Fig5.pdf]

Figure 6

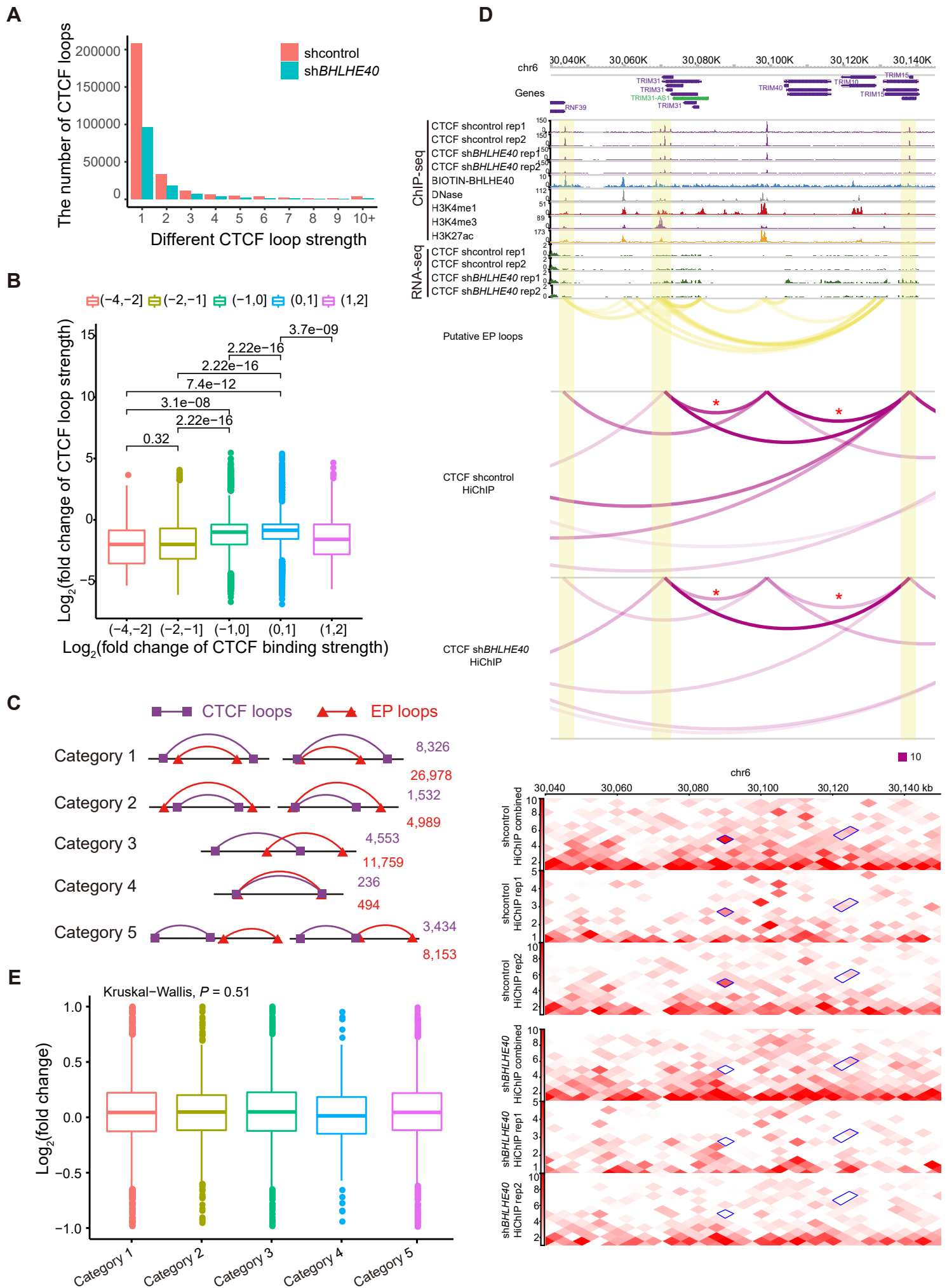

Supplement: gkaa705_Supplemental_Files [file gkaa705_supplemental_files.zip › Gongcheng_Fig6.pdf]
